# Food sources of macro- and micronutrients in young children and adults following vegan, vegetarian, and omnivorous diets

**DOI:** 10.1007/s00394-026-03898-9

**Published:** 2026-03-09

**Authors:** Venla Tilli, Topi Hovinen, Elina Kettunen, Riitta Freese, Suvi T. Itkonen, Maijaliisa Erkkola, Anu Suomalainen, Liisa Korkalo

**Affiliations:** 1 Department of Food and Nutrition, University of Helsinki, P.O. Box 66, 00014 Helsinki, Finland; 2 Research Programs Unit, Stem Cells and Metabolism, University of Helsinki, Helsinki, Finland; 3 HiLife, University of Helsinki, Helsinki, Finland; 4 HUS Diagnostics Center, Helsinki University Hospital, Helsinki, Finland

**Keywords:** Nutrient intake, Diet, Energy, Plant-based, Dairy alternative, Early childhood education and care

## Abstract

**Purpose:**

Understanding key nutrient sources in the rapidly changing food environment is essential for advising balanced food choices across different diets. We examined the main food sources of macro- and micronutrients in Finnish children and their caregivers following vegan (VGN), vegetarian or pesco-vegetarian (VGT), and omnivorous (OMN) diets.

**Methods:**

In this cross-sectional study, food consumption and nutrient intakes were analyzed from 3-day food records and compared across diet groups in 73 children aged 2–7 years (n = 29, n = 18, and n = 26 for VGN, VGT, and OMN diets, respectively) and 93 caregivers (n = 34, n = 27, and n = 32).

**Results:**

Dairy products and plant-based dairy alternatives (PBDAs), the latter often fortified, were the most significant sources of calcium, riboflavin, vitamin D and vitamin B12. In both children and adults, higher consumption of pulses, seeds, nuts and PBDAs contributed to greater intake of fiber, polyunsaturated fatty acids, and folate in the VGT and VGN groups compared to the OMN. The VGN diet was associated with a more favourable fat quality (highest intake of polyunsaturated fatty acids, lowest intake of saturated fatty acids); however, eicosapentaenoic acid (EPA) and docosahexaenoic acid (DHA) were absent from diets that contained no fish or eggs.

**Conclusion:**

Consumption of fortified dairy and PBDA products was widespread and played an important role in supporting adequate nutrition across different diet groups. Greater attention should be given to dietary sources of iodine and long-chain n-3 fatty acids.

**Supplementary Information:**

The online version contains supplementary material available at 10.1007/s00394-026-03898-9.

## Introduction

Over the past decade, plant-based diets (PBD) have gained increasing attention for their health and environmental benefits, leading to endorsements from researchers and public health authorities [1–5]. PBDs include vegan (VGN) and vegetarian (VGT) diets. A VGN diet consists exclusively of plant-based foods, whereas a VGT diets excludes meat but may include dairy products, eggs, and/or fish [5]. An omnivorous (OMN) diet imposes no dietary restrictions. While global consumption of animal-source foods continues to rise [6], a shift toward PBDs is projected to grow in high-income countries, including Finland [7, 8]. According to Healthy Finland survey by the Finnish Institute of Health and Welfare, 4.7% of the Finnish adult population followed a VGN or VGT (including lacto-ovo- and pesco-vegetarian) diets in 2022 and 2023 (personal communication, M. Simojoki, October 2025, 10.1093/eurpub/ckaf161.1366). Hereafter, we refer to vegetarian and pesco-vegetarian diets as the VGT diet.

Balanced PBDs are associated with higher intakes of fiber and polyunsaturated fatty acids (PUFA), and lower intakes of energy, saturated fatty acids (SFA), and cholesterol compared to omnivorous (OMN) diets [9]. These dietary characteristics are linked to better weight management, improved fasting blood glucose, and lower concentrations of LDL and total cholesterol [9–11]. Prospective cohort studies further suggest that adherence to PBDs is associated with a decreased risk of type 2 diabetes, certain cancers, and mortality from ischemic heart disease [10, 11]. Conversely, high consumption of red and processed meat has been associated with increased risk of obesity, type 2 diabetes, and cancer [4, 12].

Animal-source foods are central to diets in high-income countries [2]. In Finland, milk and dairy, meat, eggs, and fish contribute over 30% of energy and nearly 70% of protein intake in adults [13]. Finnish food culture is characterized by high milk and dairy consumption, which are important sources of several micronutrients, including calcium, iodine, riboflavin, and—due to a strong fortification policy, vitamin D [13, 14]. Though vitamin D fortification is voluntary practice, it is widely adopted by the food industry [15]. Although PBDs offer several health advantages, reducing the intake of animal-source foods require careful planning to ensure adequate intake of nutrients [10, 16, 17]. This is particularly important during childhood, as the unique nutritional requirements for physical growth and development add a layer of complexity to the implementation of these diets [1, 18, 19].

Currently, there is limited research on nutrient intake among individuals following VGN, VGT, and OMN diets in the contemporary food environment, where various vegan food products, including both fortified and non-fortified meat and dairy alternatives, are available. To date, only a few studies have examined food group consumption and nutrient intake across these diets [20–23]. Understanding the main food sources of nutrients is essential for developing public health strategies, establishing food-based dietary guidelines, informing dietary supplement use, and providing dietary counselling to enhance diet quality. This study aimed to identify the primary food sources of key nutrients: protein, fiber, SFA, PUFA—including linoleic acid (LA, 18:2n-6), α-linolenic acid (ALA, 18:3n-3), eicosapentaenoic acid (EPA, 20:5n-3) and docosahexaenoic acid (DHA, 22:6n-3)–, riboflavin (vitamin B2), folate, vitamin B12, vitamin D, calcium, iron, and iodine, in young children and adults following VGN, VGT, and OMN diets, and to describe the nutrient intake within these diets. These nutrients were selected based on prior literature highlighting both their positive associations with VGN diet and concerns regarding potential deficiencies [16, 24].

## Materials and methods

### Study design and participants

This study uses data from the cross-sectional MIRA2 study, described in detail elsewhere [25]. The City of Helsinki granted a research permit for the study, which was limited to Early Childhood Education and Care (ECEC) centers where at least one child had a vegan menu ordered for them. Participants included children aged 2–6 years at the time of informed consent and their caregivers. Three children turned 7 before data collection was completed, resulting in a final age range of 2–7 years.

**Fig. 1:**
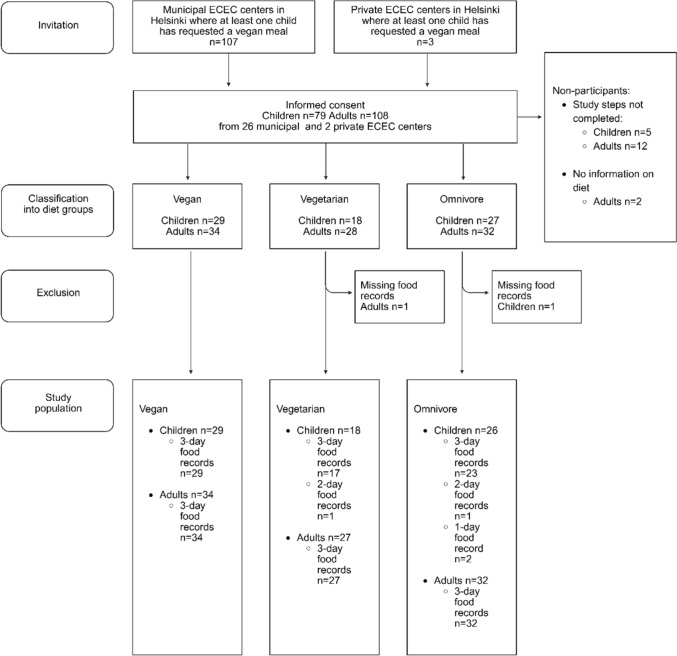
Flowchart of the participant enrolment process

The participant enrolment process is shown in Fig. 1. Recruitment was conducted via email lists administered by municipal ECEC centers. Only centers with at least one child opting for the VGN meal option were approached (n = 107 centers). Recruitment occurred in two phases: first targeting children following a VGN diet, followed by the recruitment of children following VGT and OMN diets from the same centers. Initially, only municipal ECEC centers were included; however, to ensure an adequate sample size, recruitment was expanded in March 2022 to include private ECEC centers (n = 3), applying the same criteria. Exclusion criteria for children included the following: use of oral medications (except for allergy medications), extensive food allergies, a gluten-free diet, diabetes mellitus, a body weight < 10 kg, or being breastfed during the study period. Caregivers were invited to participate regardless of their diet and were eligible if they had resided with the child for more than 50% of the time during the past year. Breastfeeding (n = 10) and pregnant (n = 4) women were included in the analysis to avoid reducing sample size, as the primary focus of this study was on food sources rather than overall nutrient adequacy.

### Sample size

The sample size was determined by recruitment feasibility. No formal power calculation was performed, as the study design relied on effect sizes identified in our previous study with 40 child participants, which revealed significant differences in nutrient intakes, nutritional status and metabolism biomarkers [18].

### Data collection

Data collection included background questionnaires, 3-day food records, and children’s anthropometric measurements. Separate questionnaires for children and caregivers were collected via the Research Electronic Data Capture (REDCap) tool (version 14.0.40, Vanderbilt University). Questionnaires were filled by the participants at home. Caregivers reported the child’s diet outside care hours and diet ordered at ECEC (8 options, detailed below in ‘*Dietary assessment’*), dietary supplement use during the past month (product, frequency and dose), and parental education level. Additional open-ended questions addressed types of salt and fat/oil used in cooking, and this information was used when entering the food record data. Caregiver questionnaire additionally covered topics on lifestyle characteristics and self-reported height and weight. Education was categorised as: basic (less than bachelor’s degree), mid (bachelor’s degree or equivalent), and high (master’s degree or higher). During a single site visit, trained research personnel measured children’s height (Seca 213 portable stadiometer), and weight (Seca 878 Mobile flat scale or Seca 704 Column scale; Seca GmbH & Co, Germany) at the University of Helsinki.

### Dietary assessment

Participants were categorized into self-identified diet groups. Diet was determined via a multiple-choice question with 8 options: (1) no special diet, (2) lactose-free or low lactose, (3) gluten-free, (4) excluding red meat, (5) vegan (excluding all animal-sourced products), (6) vegetarian (excluding meat but including fish, eggs, and/or dairy), (7) restricted for religious reasons, and (8) other, with an open-text field for specification. Options 1–4 and 8 were classified as the OMN group using the open-text information for clarification if needed, option 6 as the VGT group, and option 5 as the VGN group. No children were reported to follow a gluten-free diet as specified in the exclusion criteria, and no responses were recorded for option 7. Participants were assigned to OMN, VGT, or VGN groups based on this parent/self-reported diet or inferred from food records if such information was lacking (n = 5). For VGT participants, the use of eggs, dairy, fish, and seafood was further clarified with an additional question. In case caregivers reported different diets for the child at home and at ECEC, the least restrictive diet for the child was assigned.

Food records were available for 73 children and 93 adults. Food record days (two weekdays and one weekend day) were assigned by research personnel. These days were not always consecutive days, and the families had a possibility to change the dates if the assigned days were unsuitable to them (e.g., due to travel or illness). Caregivers recorded child’s food intake at home, while ECEC staff did so during care hours on a separate form, both for the same days. Food consumption was documented using validated food picture booklets [26, 27] to estimate portion sizes. The research team resolved inconsistencies in food records by requesting clarification during the site visit or via telephone. Four children had incomplete food records, mainly due to unreported daycare meals. Therefore, only one day of complete data was available for two OMN participants, and two days for one OMN and one VGT participant. Municipal food services provided recipes and information about the food products used at ECEC centers.

Food records were entered into Aromi Diet Software (CGI Inc. Version 14.10.14), linked to the National Food Composition Database (Fineli®, Release 20). New products and recipes were manually added based on nutrient labels and ingredient lists provided by manufacturers and recipe compilation by the research team when no matching items were available in the Fineli database. After data entry, composite dishes were disaggregated and ingredient-level data was exported and categorized into 20 different groups according to the Global Individual Food consumption data Tool (GIFT) [28], with minor adjustments. The analysis was performed using the main group classification, except for plant-based dairy alternatives (PBDAs), which were classified as their own group from the main groups of ‘cereals’, ‘roots and tubers’, and ‘pulses, nuts, and seeds’ (see Supplementary Appendix [SA] Table S1). The groups ‘foods for particular nutritional uses’, ‘supplementary foods’ and ‘food additives’ were merged into a single group labelled ‘other’. Extensive checks were performed on food records by examining extreme values of nutrient intakes and unusual food volumes consumed, separately for children and adults. Nutrient intakes calculated at the ingredient level reflect raw forms and do not account for cooking-related nutrient losses.

Individual daily food consumption and nutrient intakes were calculated as the mean of recorded days. To adjust for energy intake, daily intake values were divided by total energy (excluding energy from alcohol in adults), resulting in average intake per megajoule (MJ). Group-level intakes were derived from individual means [29]. Absolute food group intakes are presented in the SA [Table S2]. Percentages of energy intake (E%) were calculated for carbohydrates, protein, fat, PUFA, monounsaturated fatty acids (MUFA) and SFA. Main food sources and intakes for protein, fiber, riboflavin, folate, vitamin B12, vitamin D, calcium, iron, iodine, and fatty acids (SFA, PUFA, LA, ALA, EPA, and DHA) are reported here; intakes of other nutrients are provided in the SA [Table S3]. Food group contributions to nutrient intake are expressed as percentages, available in the SA [TablesS4–S34]. Consistent with Finnish nutrition policy, which emphasizes meeting nutrient needs primarily through foods rather than supplements, we excluded nutrient intake from dietary supplements in this analysis.

### Statistical analyses

Statistical analyses were performed in R Studio (version 2024.12.0; Posit Software, PBC, Boston, MA). Differences in food consumption and nutrient intakes between diet groups separately in children and adults were assessed with the non-parametric Kruskal–Wallis test. Post hoc pairwise comparisons were conducted using two-tailed Wilcoxon Rank Sum Test, with *p*-values corrected for multiple testing using the Benjamini–Hochberg False Discovery Rate method. Such corrected *p*-value of < 0.05 was considered statistically significant. For the consumption of ‘milk and dairy’, ‘eggs’, ‘fish’, and ‘meat’, pairwise comparisons were conducted only between the OMN and VGT groups, as consumption in the VGN group was expected to be zero.

## Results

### Participant characteristics

The mean age was 4.1 years (SD 1.4) for children, and 37.9 years (4.3) for adults (Table 1). Among the children, there were 10 sibling pairs. 6% of VGT children and 4% of VGT adults were categorized as lacto-vegetarians, 28% and 37% as lacto-ovo vegetarians, and 67% and 59% as pesco-vegetarians. One (3%) OMN adult reported consuming no red meat. Majority of both children and adults were classified as having normal weight based on BMI reference values [30, 31]. Vitamin D supplement use was common across all diet groups, and all children in the VGN group consumed vitamin B12 supplements (Table 1). Four women were pregnant, and 10 women were breastfeeding. Adult participants were generally well educated.

**Table 1:** Participant characteristics

	Children			Adults		
	OMN	VGT	VGN	OMN	VGT	VGN
N (Female, %)	26 (48%)	18 (50%)	29 (59%)	32 (59%)	27 (59%)	34 (71%)
Age, years, mean (SD)	4.1 (1.3)	4.2 (1.6)	4.1 (1.5)	37.5 (4.6)	39.7 (4.3)	36.8 (3.4)
*(ISO-)BMI,* *kg/m*^*2*^, *mean* *(SD)*	21.8 (3.9)	23.1 (3.8)	21.7 (3.5)	23.9 (2.8)	24.4 (3.7)	23.8 (4.4)
Underweight (BMI < 18.5 kg/m^2^), n (%)	6 (23%)	3 (17%)	2 (7%)	0 (0%)	1 (4%)	1 (3%)
Normal weight (BMI 18.5–25 kg/m^2^), n (%)	12 (46%)	9 (50%)	23 (79%)	21 (66%)	12 (44%)	24 (71%)
Overweight or obese (BMI > 25 kg/m^2^), n (%)	6 (23%)	6 (33%)	4 (14%)	9 (28%)	12 (44%)	9 (26%)
Missing values, n (%)	2 (8%)	0 (0%)	0 (0%)	2 (6%)	2 (7%)	0 (0%)
Mother’s diet, OMN: VGT: VGN, %^a^	87: 13: 0	0: 100: 0	0: 0: 100			
Father’s diet, OMN: VGT: VGN, % ^a^	100: 0: 0	17: 83: 0	13: 13: 73			
*Dietary supplement use,* *yes,* *n(%)*^*b*^						
Vitamin D	23 (88%)	18 (100%)	29 (100%)	24 (75%)	20 (74%)	32 (94%)
Vitamin B12	7 (27%)	9 (50%)	29 (100%)	8 (25%)	13 (48%)	31 (97%)
Iodine	5 (19%)	7 (39%)	26 (90%)	6 (19%)	7 (26%)	28 (82%)
N-3 fatty acids	2 (8%)	3 (17%)	4 (14%)	3 (9%)	5 (19%)	4 (12%)
Missing values, n (%)	1 (4%)	0 (0%)	0 (0%)	2 (6%)	2 (7%)	0 (0%)
*Educational level*^*c*^						
Basic level education				3 (9%)	0 (0%)	4 (12%)
Mid-level education				11 (34%)	5 (19%)	7 (21%)
High-level education				16 (50%)	20 (74%)	23 (68%)
Pregnant, yes, n (%)^d^				2 (11%)	2 (12%)	0 (0%)
Breastfeeding, yes, n (%)^d^				4 (21%)	1 (6%)	5 (21%)

OMN, omnivorous diet; VGT, vegetarian diet; VGN, vegan diet

BMI, body-mass-index. Age and sex-adjusted ISO-BMI for children was calculated according to Finnish health information online service Terveyskirjasto (Saari A. www.terveyskirjasto.fi, 2024). BMI for adults was calculated according to Finnish health information online service Terveyskirjasto (Pelttari H. www.terveyskirjasto.fi, 2024)

^a^Parental diet information is available only for participating parents

^b^Reported use of dietary supplements in the background questionnaire. ‘Yes’ indicates use of any supplement containing the nutrient of interest

^c^Missing values from n = 2 OMN and n = 2 VGT adults

^d^Percentages calculated within the female population. Missing values from n = 1 OMN adult

### Food group consumption

**Children**: Consumption (g/MJ) of ‘pulses, seeds, and nuts’ was significantly higher in the VGN and VGT groups compared to the OMN (Table 2). The combined intake of ‘fruits and vegetables’ was lowest in the OMN group. Consumption (g/MJ) of ‘PBDAs’ was highest in the VGN group, while ‘milk and dairy’ consumption was highest in the OMN group. There were no significant differences in the consumption of ‘fish’ and ‘eggs’ between the VGT and OMN groups.

**Table 2:** Food group consumption as per megajoule in children and adults

	Children				Adults			
	OMN	VGT	VGN		OMN	VGT	VGN	
Food group, g/MJ	n = 26	n = 18	n = 29	*p*-value	n = 32	n = 27	n = 34	*p*-value
Cereals	25	25	27	0.409	23	27	25	0.056
	[19–30]	[20–28]	[23–32]		[18–27]	[23–31]	[22–29]	
Roots, tubers	7.8	8.3	6.6	0.485	8.1	3.9	6.5	0.340
	[4.8–15.2]	[3.3–11.4]	[3.7–9.1]		[2.8–10.1]	[1.0–9.7]	[3.7–10.3]	
Pulses, seeds, nuts	1.4^a^	7.8^b^	11.4^b^	< 0.001	2.3^a^	15.7^b^	18.0^b^	< 0.001
	[0.2–3.4]	[5.4–13.3]	[8.8–13.7]		[0.5–6.7]	[6.7–21]	[15.3–22.7]	
Plant-based dairy alternatives (PBDA)	0^a^	41^b^	86^c^	< 0.001	2^a^	28^b^	46^c^	< 0.001
	[0–8]	[20–63]	[67–107]		[0–12]	[16–32]	[30–58]	
Milk, dairy^d^	69^a^	38^b^	0	0.041	33^a^	13^b^	0	< 0.001
	[58–108]	[15–63]	[0–0]		[20–50]	[5–15]	[0–0]	
Sum of milk, dairy, PBDAs	82	90	86	0.984	38	35	46	0.428
	[61–117]	[73–103]	[67–107]		[30–54]	[29–52]	[30–58]	
Eggs^d^	1.9	0.5	0	0.074	1.7	0.4	0	0.378
	[0.7–4.7]	[0–1.5]	[0–0]		[0.5–3.5]	[0–4.4]	[0–0]	
Fish^d^	4.3	0.5	0	0.100	4.3^a^	0^b^	0	0.004
	[0–8.6]	[0–4.4]	[0–0]		[1.0–6.7]	[0–1.9]	[0–0]	
Meat^d^	9^a^	0^b^	0	< 0.001	12.5^a^	0^b^	0	< 0.001
	[6.1–13.7]	[0–0]	[0–0]		[7.4–20]	[0–0]	[0–0]	
Vegetables	20	19	18	0.417	24^a^	34^b^	28^a,b^	0.003
	[14–26]	[15–33]	[14–21]		[16–31]	[28–53]	[22–37]	
Fruits	24	31	31	0.145	14^a^	14^a,b^	21^b^	0.044
	[13–35]	[23–39]	[25–37]		[6–20]	[9–24]	[10–33]	
Sum of fruits, vegetables	40^a^	58^b^	48^a,b^	0.031	39^a^	53^b^	47^b^	0.009
	[33–55]	[48–74]	[39–59]		[27–52]	[39–66]	[42–67]	
Fats, oils	3.7	3.4	3.9	0.444	3.1	2.5	4.5	0.385
	[2.2–4.7]	[2.4–4]	[2.9–4.9]		[2–3.9]	[1.9–4.5]	[2.2–5.4]	
Sweets, sugars	2.7	4.8	3.9	0.579	4.6	4	3.4	0.784
	[1.6–6.3]	[1.9–6.3]	[2.1–5.5]		[1.6–7.5]	[2.1–6.4]	[2.4–5.6]	
Spices, condiments	1.1	1.3	1.3	0.426	1.9	2.1	2.3	0.663
	[0.3–1.7]	[0.8–1.6]	[0.7–2.5]		[0.8–3.2]	[1.4–3.6]	[1.2–3.9]	
Beverages	33	40	45	0.183	97	94	97	0.714
	[21–47]	[28–45]	[32–59]		[82–128]	[69–132]	[47–123]	
Savory snacks	0	0	0	0.364	0^a^	0^b^	0^a,b^	0.022
	[0–0]	[0–0]	[0–0]		[0–0]	[0–1.8]	[0–1.2]	
Other^e^	0.1	0.2	0.4	0.084	0.1	0.1	0.3	0.058
	[0–0.4]	[0–0.6]	[0.1–0.6]		[0–0.3]	[0–0.3]	[0.1–0.5]	

OMN, omnivorous diet; VGT, vegetarian diet; VGN, vegan diet

Data are presented as medians and interquartile ranges [25th–75th percentiles]

Group differences were assessed using the Kruskal–Wallis test. For variables with *p* < 0.05, pairwise comparisons were performed using the Wilcoxon rank-sum test. *P*-values were adjusted for multiple testing using the Benjamini–Hochberg false discovery rate method. Statistically significant differences (adjusted *p* < 0.05) are indicated by different superscript letters (a, b, c), i.e. medians not sharing a common superscript letter are significantly different from each other

^d^For these food groups, only pairwise comparisons between OMN and VGT groups were assessed

^e^Food group ‘Other’ includes: foods for particular nutritional uses (baby foods (porridges), protein powders, protein bars, protein shakes); nutritional yeast, vegemite, psyllium; food additives (sweeteners, lifting agents, baking yeast)

**Adults**: As in children, similar observations were made for the consumption (g/MJ) of ‘pulses, seeds, and nuts’, ‘milk and dairy’ and ‘PBDAs’ in adults (Table 2). The combined intake of ‘fruits and vegetables’ was higher in the VGN and VGT groups compared to the OMN group. ‘Fish’ consumption was highest in the OMN group, while one participant in the VGT group reported consuming ‘meat’.

**General**: Withing the group ‘PBDAs’, 84% of the reported *drinks and yogurts* were fortified with calcium (most commonly 120 mg/100 mL), 69% with riboflavin (0.21 mg/100 mL), 79% with vitamin B12 (0.38 µg/100 mL), 77% with vitamin D (0.75 µg/100 mL), and 19% with folate and/or iodine (30 µg and 22.5 µg/100 mL, respectively). Furthermore, *‘drinks and yogurts’* accounted for 94% of total PBDA consumption (g/d) and contributed 80% of the total energy intake from this food group.

### Energy and macronutrient intake and main sources

**Fig. 2:**
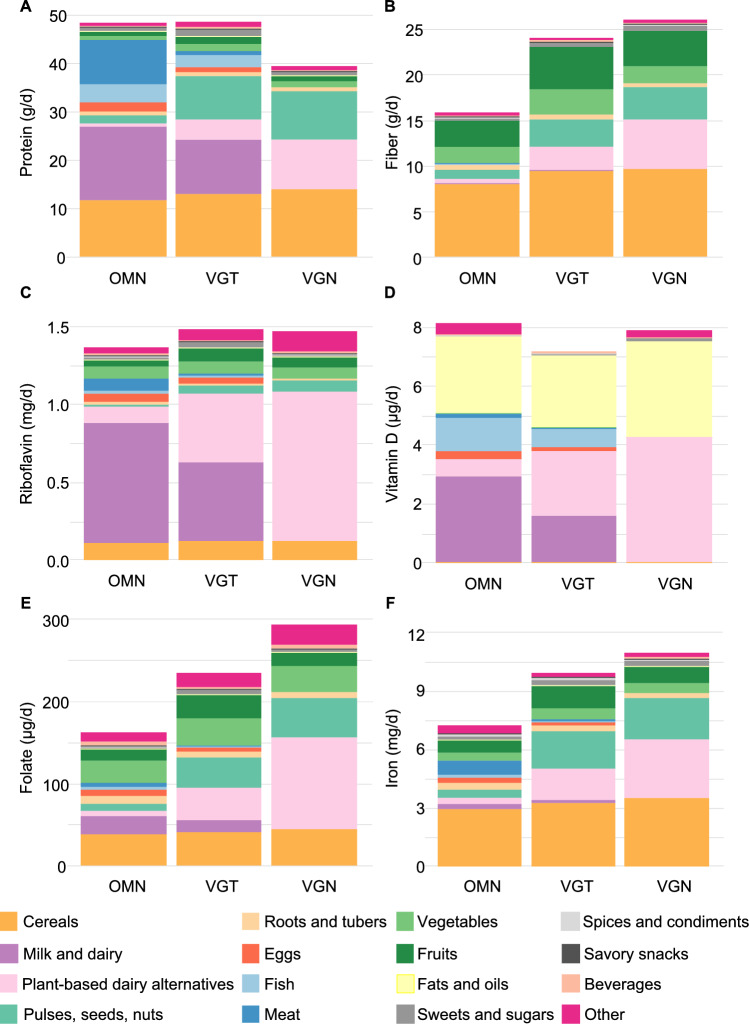
Mean intake of (**A**) protein, (**B**) fiber, (**C**) riboflavin, (**D**) vitamin D, (**E**) folate, and (**F**) iron from different food sources in children following omnivorous (OMN), vegetarian (VGT), and vegan (VGN) diets

**Children**: The OMN group had the lowest energy intake (4.6 MJ; Table 3). Protein (E%) intake was lowest in the VGN and highest in the OMN group (11 vs. 16 E%, respectively; Table 3). Key sources of energy and protein for all diet groups included ‘cereals’, ‘milk and dairy products’, and/or ‘PBDAs’ (Fig. 2A). ‘Pulses, seeds, and nuts’ accounted for 25% (VGN) and 18% (VGT) of protein intake, while meat accounted for 19% of the intake in the OMN group [SA; Table S5]. No difference in fat (E%) intake was observed (Table 3). Intake of carbohydrates (53 E%) and fiber (15 g/d, 3.1 g/MJ) were lowest in the OMN group. Higher consumption of ‘pulses, seeds, and nuts’ and ‘PBDAs’ contributed to the higher fiber intake observed in VGT and VGN groups (Fig. 2B).

**Table 3:** Nutrient intakes by diet groups in children and adults

	Children				Adults			
	OMN	VGT	VGN		OMN	VGT	VGN	
Nutrient	n = 26	n = 18	n = 29	*p*-value	n = 32	n = 27	n = 34	*p*-value
Daily energy, MJ	4.6^a^	5.7^b^	5.2^b^	0.019	8.3	8.5	8.4	0.887
	[4.3–5.6]	[5.1–6.4]	[4.9–6.6]		[7.5–9.3]	[7.2–9.7]	[7.6–10]	
Protein, E%	16^a^	14^b^	11^c^	< 0.001	17^a^	14^b^	14^b^	< 0.001
	[14–18]	[14, 15]	[11–13]		[16–19]	[13–16]	[12–15]	
*g/kg body weight*	2.8^a^	2.5^a,b^	2.2^b^	0.030	1.2^a^	1.0^b^	0.9^b^	0.005
	[2.5–3.1]	[2.1–2.7]	[1.9–2.7]		[0.9–1.6]	[0.9–1.1]	[0.8–1.1]	
*g/d*	45^a^	49^a^	40^b^	0.003	85^a^	72^a,b^	64^b^	0.001
	[39–56]	[41–55]	[33–45]		[68–101]	[58–87]	[57–81]	
*g/MJ*	9.3^a^	8.2^b^	6.7^c^	< 0.001	10^a^	8.4^b^	8^b^	< 0.001
	[8.4–10.6]	[8–8.7]	[6.5–7.4]		[9.5–11.2]	[7.8–9.3]	[6.8–8.9]	
Carbohydrate, E%	53^a^	53^a,b^	57^b^	0.025	44^a^	46^a,b^	47^b^	0.004
	[48–55]	[50–56]	[52–60]		[39–48]	[43–51]	[45–53]	
*g/d*	137^a^	173^b^	166^b^	0.011	188^a^	206^a,b^	225^b^	0.036
	[126–163]	[150–187]	[148–201]		[160–229]	[189–246]	[200–262]	
*g/MJ*	30	30	31	0.076	24^a^	25^a,b^	26^b^	0.039
	[27–31]	[28–31]	[29–33]		[21–27]	[24–28]	[24–29]	
Fiber, g/d	15^a^	24^b^	26^b^	< 0.001	21^a^	33^b^	39^b^	< 0.001
	[14–18]	[20–29]	[23–30]		[17–28]	[28–41]	[32–46]	
*g/MJ*	3.1^a^	4.1^b^	4.6^b^	< 0.001	2.6^a^	3.9^b^	4.3^b^	< 0.001
	[2.8–3.7]	[3.6–4.8]	[4.3–4.9]		[2.1–3.1]	[3.3–4.7]	[3.8–4.7]	
Fat, E%	32	32	32	0.995	38	38	38	0.633
	[30–34]	[30–33]	[30–36]		[36–42]	[35–43]	[35–42]	
*g/d*	41	48	47	0.130	90	93	80	0.892
	[35–54]	[43–56]	[40–55]		[71–106]	[67–111]	[69–114]	
*g/MJ*	8.7	8.7	8.6	0.995	10.2	10.3	10.3	0.633
	[8.1–9.3]	[8.2–8.9]	[8–9.7]		[9.7–11.5]	[9.5–11.5]	[9.4–11.4]	
Saturated fatty acids, E%	11^a^	9^a^	7^b^	< 0.001	14^a^	12^b^	9^c^	< 0.001
	[9–13]	[8–11]	[6–8]		[12–16]	[8–14]	[7–10]	
*g/d*	15^a^	13^a^	10^b^	< 0.001	31^a^	27^a^	19^b^	< 0.001
	[11–18]	[12–19]	[8–12]		[24–40]	[18–37]	[14–27]	
*g/MJ*	2.9^a^	2.4^a^	1.8^b^	< 0.001	3.8^a^	3.2^b^	2.4^c^	< 0.001
	[2.5–3.5]	[2.1–3.1]	[1.6–2.1]		[3.2–4.4]	[2.2–3.9]	[1.9–2.7]	
Monounsaturated fatty acids, E%	12	12	13	0.613	14	14	15	0.949
	[11–14]	[11–13]	[11–14]		[13–17]	[13–17]	[13–16]	
*g/d*	15	18	18	0.171	35	36	31	0.967
	[13–20]	[17–21]	[15–22]		[27–39]	[25–40]	[26–43]	
*g/MJ*	3.4	3.2	3.4	0.613	3.9	3.8	3.9	0.949
	[3–3.7]	[3–3.5]	[3.1–3.7]		[3.5–4.6]	[3.4–4.6]	[3.5–4.4]	
Polyunsaturated fatty acids, E%	6^a^	8^b^	10^c^	< 0.001	7^a^	9^b^	10^c^	< 0.001
	[5–8]	[6–8]	[8–11]		[6–8]	[8–10]	[9–12]	
*g/d*	8^a^	11^b^	15^c^	< 0.001	15^a^	19^b^	23^c^	< 0.001
	[7–9]	[10–14]	[12–17]		[13–19]	[17–22]	[19–32]	
*g/MJ*	1.6^a^	2.1^b^	2.7^c^	< 0.001	1.8^a^	2.4^b^	2.8^c^	< 0.001
	[1.4–2.0]	[1.7–2.3]	[2.3–3]		[1.6–2.0]	[2.1–2.6]	[2.6–3.3]	
Riboflavin, mg/d	1.3	1.5	1.5	0.581	1.8	1.5	1.7	0.806
	[1.0–1.7]	[1.2–1.7]	[1.1–1.7]		[1.2–2.1]	[1.3–1.9]	[1.3–2.0]	
*mg/MJ*	0.26	0.25	0.25	0.847	0.20	0.19	0.19	0.863
	[0.24–0.31]	[0.21–0.3]	[0.21–0.3]		[0.17–0.22]	[0.17–0.22]	[0.16–0.24]	
Folate, µg/d	157^a^	230^b^	287^c^	< 0.001	262^a^	374^b^	456^c^	< 0.001
	[138–180]	[219–238]	[219–343]		[230–306]	[308–492]	[393–527]	
*µg/MJ*	31^a^	40^b^	53^c^	< 0.001	31^a^	42^b^	56^c^	< 0.001
	[25–37]	[33–46]	[43–61]		[26–36]	[36–57]	[46–61]	
Vitamin B12, µg/d	3.1^a^	2.7^a,b^	2.2^b^	0.021	5.5^a^	2.9^b^	1.9^c^	< 0.001
	[2.5–4]	[1.5–3.6]	[1.6–3]		[3.3–7.2]	[1.6–4.3]	[1.3–2.8]	
*µg/MJ*	0.6^a^	0.4^b^	0.4^b^	0.001	0.7^a^	0.3^b^	0.2^c^	< 0.001
	[0.5–0.8]	[0.2–0.6]	[0.3–0.5]		[0.5–0.8]	[0.2–0.5]	[0.2–0.3]	
Vitamin D, µg/d	7.3	6.5	7.5	0.641	8.6	6.1	6.8	0.045
	[5.5–10.7]	[5.3–9.6]	[6.5–9.2]		[5.6–10.6]	[3.4–9.2]	[4.4–8.5]	
*µg/MJ*	1.5	1.4	1.5	0.131	1^a^	0.7^b^	0.7^a,b^	0.028
	[1.2–2.1]	[0.9–1.6]	[1.1–1.7]		[0.7–1.3]	[0.5–1]	[0.6–1]	
Calcium, mg/d	745	825	748	0.525	1010	1010	920	0.467
	[597–990]	[723–967]	[590–910]		[703–1270]	[858–1250]	[730–1150]	
*mg/MJ*	160	145	126	0.205	125	126	106	0.308
	[122–189]	[127–182]	[110–156]		[99–138]	[110–150]	[87–137]	
Iodine, µg/d	155^a^	135^a^	98^b^	0.001	215^a^	168^b^	109^c^	< 0.001
	[113–180]	[115–182]	[82–125]		[178–254]	[132–189]	[87–152]	
*µg/MJ*	30^a^	26^b^	17^c^	< 0.001	26^a^	18^b^	14^c^	< 0.001
	[26–34]	[21–30]	[13–22]		[23–31]	[15–24]	[12–15]	
Iron, mg/d	6.8^a^	9.5^b^	10.8^b^	< 0.001	11.1^a^	14.8^b^	17.6^b^	< 0.001
	[6.1–8]	[7.7–11.8]	[8.9–12.6]		[9.7–12.5]	[12.5–18.4]	[14.6–20.5]	
*mg/MJ*	1.4^a^	1.8^a,b^	1.9^b^	< 0.001	1.4^a^	1.9^b^	2^c^	< 0.001
	[1.2–1.5]	[1.4–2]	[1.8–2.1]		[1.2–1.5]	[1.5–2.1]	[1.8–2.4]	
Linoleic acid (LA, 18:2n-6), g/d	4.7^a^	7.1^b^	10.3^c^	< 0.001	10.2^a^	14.7^b^	17.9^b^	< 0.001
	[3.8–6.6]	[6.3–8.4]	[8.0–13.1]		[7.8–12.7]	[12.5–17.2]	[13.3–23.3]	
*g/MJ*	0.9^a^	1.3^b^	1.9^c^	< 0.001	1.2^a^	1.7^b^	2.1^c^	< 0.001
	[0.8–1.2]	[1.0–1.5]	[1.6–2.1]		[0.9–1.4]	[1.5–2.1]	[1.7–2.4]	
α–Linolenic acid (ALA, 18:3n-3), g/d	1.3^a^	2.0^b^	2.5^b^	< 0.001	2.4^a^	3.3^b^	4.6^c^	< 0.001
	[0.9–1.7]	[1.7–2.6]	[1.8–3.5]		[1.8–3.2]	[2.5–4.3]	[3.1–6.4]	
*g/MJ*	0.3^a^	0.3^b^	0.4^b^	< 0.001	0.3^a^	0.4^b^	0.6^c^	< 0.001
	[0.2–0.3]	[0.3–0.5]	[0.4–0.5]		[0.2–0.4]	[0.3–0.5]	[0.4–0.6]	
Eicosapentaenoic acid (EPA, 20:5n-3), mg/d	18.8^a^	2.9^b^	0^c^	< 0.001	165^a^	0^b^	0^c^	< 0.001
	[6.4–85.8]	[0–52.9]	[0–0]		[29.4–288]	[0–25.4]	[0–0]	
*mg/MJ*	4.5^a^	0.5^b^	0^c^	< 0.001	19.2^a^	0^b^	0^c^	< 0.001
	[1.2–19.4]	[0–10.6]	[0–0]		[3.4–30.6]	[0–3.3]	[0–0]	
Docosahexaenoic acid (DHA, 22:6n-3), mg/d	66.2^a^	13^b^	0^c^	< 0.001	431^a^	39.2^b^	0^c^	< 0.001
	[24.6–254]	[1.8–171]	[0–0]		[57–897]	[7.9–94.6]	[0–0.3]	
*mg/MJ*	16.2^a^	2.36^b^	0^c^	< 0.001	54.2^a^	5.22^b^	0^c^	< 0.001
	[6.2–49.8]	[0.3–33.8]	[0–0]		[8.4–94]	[0.9–12.5]	[0–0]	

OMN, omnivorous diet; VGT, vegetarian diet; VGN, vegan diet

Data are presented as medians and interquartile ranges [25th–75th percentiles]

Group differences were assessed using the Kruskal–Wallis test. For variables with *p* < 0.05, pairwise comparisons were performed using the Wilcoxon rank-sum test. *P*-values were adjusted for multiple testing using the Benjamini–Hochberg false discovery rate method. Statistically significant differences (adjusted *p* < 0.05) are indicated by different superscript letters (a, b, c), i.e. medians not sharing a common superscript letter are significantly different from each other

**Fig. 3:**
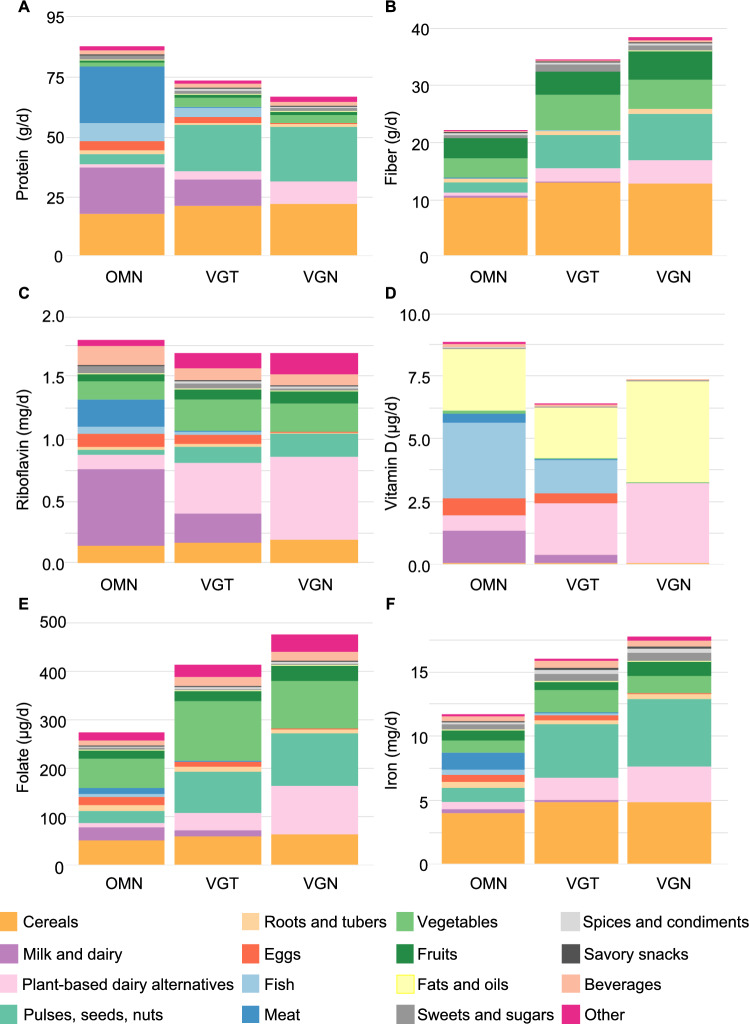
Mean intake of (**A**) protein, (**B**) fiber, (**C**) riboflavin, (**D**) vitamin D, (**E**) folate, and (**F**) iron from different food sources in adults following omnivorous (OMN), vegetarian (VGT), and vegan (VGN) diets

**Adults**: No difference in energy or fat (E%) intake were observed (Table 3). Protein (E%) intake was highest in the OMN group (Fig. 3A), where ‘meat’ was the primary source and accounted for 26% of intake. In contrast, nearly 70% of protein intake in the VGN group was obtained from ‘pulses, seeds, nuts’ and ‘cereals’ [SA; Table S5]. Intake of carbohydrates (44 E%) and fiber (21 g/d, 2.6 g/MJ) were lowest in the OMN group (Table 3).

### Micronutrient intakes and main sources

**Children**: No differences in riboflavin, vitamin D, or calcium intakes were observed (Table 3). ‘Milk and dairy’ and/or ‘PBDAs’ were the main sources of riboflavin (Fig. 2C) and calcium [SA; Table S23]. Vitamin D was also obtained from these foods, in addition to ‘fats and oils’ across diet groups, and ‘fish’ in the VGT and OMN groups (Fig. 2D). Folate intake was highest in the VGN and lowest in the OMN group (287 vs. 157 µg/d, respectively; Table 3), primarily due to higher consumption of ‘PBDAs’ and ‘pulses, seeds, and nuts’ in the VGN group (Fig. 2E).

In the VGN group, 91% of vitamin B12 intake was obtained from ‘PBDAs’, with the remainder sourced from products like nutritional yeast (classified under ‘Other’) [SA; Table S17]. Cereals were the main source of iron, and higher intakes in the VGN and VGT groups were due to greater consumption of ‘PBDAs’ and ‘pulses, seeds, and nuts’ (Fig. 2F). Iodine intake was lowest in the VGN and highest in the OMN group (98 vs. 155 µg/d, respectively; Table 3). Key sources included ‘milk and dairy’, ‘PBDAs’, ‘cereals’, and approximately 20% from ‘spices and condiments’ (mainly iodized salt) across diet groups [SA; Table S24].

**Adults**: As in children, no differences in riboflavin or calcium intakes were observed (Table 3). ‘Milk and dairy’, and/or ‘PBDAs’ were the primary sources for these nutrients, with ‘pulses, seeds, and nuts’ contributing 26% of calcium intake in the VGN group [SA; Tables S13, S23]. ‘Fish’ contributed 34% (OMN) and 21% (VGT) of vitamin D intake, while 98% of the intake was sourced from ‘PBDAs’ and ‘fats and oils’ in the VGN group [SA; Table S20].

Folate intake was highest in the VGN and lowest in the OMN group (456 vs. 262 µg/d, respectively), whereas iodine intake showed the opposite trend (109 vs. 215 µg/d; Table 3). Trends in vitamin B12 and iron intakes were similar to those observed in children (Table 3). In the VGN group, 30% of iron intake was obtained from ‘pulses, seeds, and nuts’ [SA; Table S25].

### Fatty acid intake and main sources

**Fig. 4:**
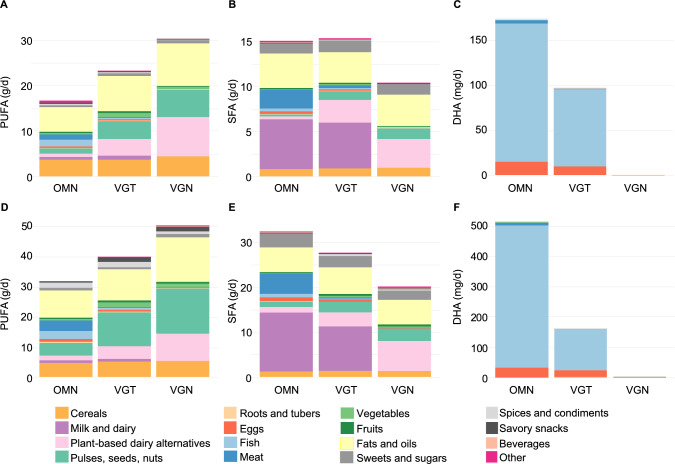
Mean intake of polyunsaturated fatty acids (PUFA), saturated fatty acids (SFA), and docosahexaenoic acid (DHA) from different food sources in children (**A**–**C**) and adults (**D**–**F**) following omnivorous (OMN), vegetarian (VGT), and vegan (VGN) diets

**Children**: PUFA intake was highest in the VGN and lowest in the OMN group (10 vs. 6 E%, respectively; Table 3). In contrast, SFA intake was highest in the VGT and OMN groups, due to ‘milk and dairy’, and ‘meat’ consumption (the latter only in OMNs) (Fig. 4B). Key sources of LA and ALA were ‘cereals’ and ‘fats and oils’ across diet groups, with higher intakes in the VGN and VGT groups supported by greater consumption of ‘pulses, seeds, and nuts’ and ‘PBDAs’ [SA; Tables S31, S32]. Intakes of long-chain n-3 fatty acids EPA and DHA were zero in the VGN group, as these were exclusively sourced from ‘fish’ and ‘eggs’, consumed only in the OMN and VGT groups (Fig. 4C).

**Adults**: Fatty acid intakes and main sources in adults mirrored those observed in children.

## Discussion

In this study, we found that pulses, seeds, nuts and PBDA were important sources of several key nutrients—such as protein, PUFA, folate and iron—in the diets of VGT and VGN Finnish children and adults. Our findings highlight the critical role of food fortification in supporting adequate intake of riboflavin, vitamin B12, calcium, and iodine in individuals following these diets. Furthermore, vitamin D fortification effectively supports adequate intake across all diet groups. In the diets of OMN Finnish children and adults, animal-source foods were major contributors to energy, protein, and several micronutrients, including vitamin B12, vitamin D, riboflavin, calcium, iodine, EPA, and DHA. Notably, EPA and DHA were absent in diets that excluded fish and eggs.

### Macronutrients and fiber

On average, children and adults across diet groups met the Nordic Nutrition Recommendations (NNR) 2023 for protein (E%) intake (recommended intake [RI], 10–20 E% for both children and adults) [1]. However, protein intake was lower in the VGN group compared to the OMN group, primarily due to the contribution of animal-source protein in the latter. Replacing animal protein sources—such as milk, meat, eggs, and fish—with plant-based alternatives raises concerns about protein quality and bioavailability, as plant proteins may contain antinutritional compounds that reduce bioavailability, and are limited in certain amino acids, including lysine, tryptophan (in cereals), methionine, and cysteine (in pulses) [32]. Still, increasing plant protein intake while reducing animal protein is considered beneficial, due to health-promoting components such as PUFAs and fiber present in plant sources (e.g., legumes) [33, 34]. A systematic review and meta-analysis found a positive association between higher total and animal protein intake and increased BMI, while low protein intake was not shown to impair growth in well-nourished Western children [35]. Previous studies have similarly reported lower protein content in PBDs compared to OMN diets [16, 24].

Average intakes of carbohydrates (RI for all 45–60 E%) and fiber (RI 2–3 g/MJ for children and ≥ 3 g/MJ for adults) were within the RI´s for all child groups as well as the adult VGN and VGT groups, but less than the RI´s in the adult OMN group. According to the FinDiet 2017 Study, approximately 70% of Finnish adults have inadequate carbohydrate intake, and only 11% of adult men and 2% of women meet the RI for fiber [13]. Similar challenges in the intake of carbohydrates have been reported in other Nordic countries [36]. In our study, the main source of carbohydrates was cereals, while on average fruit and vegetable consumption did not reach the RI of 500–800 g/d in any adult group [37]. PBDs, characterized by higher consumption of pulses, seeds, nuts, and vegetables, are associated with greater fiber intake [22]. Thus, the inclusion of these fiber-rich foods such as pulses, seeds, and nuts could substantially improve fiber intake in individuals following OMN diets.

Fat quality was more favourable in the VGN group among children and adults, as characterized by lowest intake of SFA (E%) and highest intake of PUFA (E%). To improve dietary fat quality, individuals following OMN diets could benefit from partially replacing foods containing animal fat with plant-source foods rich in MUFAs and PUFAs, such as vegetable oils, nuts, seeds, and pulses. Essential fatty acids—LA and ALA—were primarily obtained from such plant sources. In contrast, long-chain n-3 fatty acids, EPA and DHA, were obtained exclusively from animal-source foods, particularly fish and, to a lesser extent, eggs. Consequently, children and adults following a VGN diet, as well as other participants with low or no fish consumption, had negligible intakes of EPA and DHA. Current Finnish National Nutrition Recommendations advice a daily n-3 PUFA intake of 1 E%, and it is assumed that sufficient ALA intake supports adequate DHA status in vegans [37]. The recommendations include no guidance on long-chain n-3 fatty acid supplement use. However, the long-term health implications of chronically low intakes of DHA among VGN and some VGT populations remains unclear. This concern may be particularly relevant during pregnancy and early childhood, a critical period in which DHA plays a vital role in brain and retinal development [38].

### Vitamin D and iodine

PBDAs, the majority of which were fortified, contributed significantly to vitamin D and iodine intake in VGN and VGT diets—similar to milk and dairy products in OMN diets, particularly among children. In Finland, most fluid milk products are fortified with vitamin D [15], as natural sources are mainly limited to fatty fish, fish liver oil, and to a lesser extent, egg yolk [39]. National voluntary fortification practices include the addition of vitamin D_3_ to fluid milk products (1 µg/100 mL) and fat spreads (20 µg/100 g) [14]. The Finnish National Nutrition Recommendations advice the use of fortified PBDAs as alternatives for dairy products [37]. To bridge nutritional gaps between plant-based and animal-source foods, many PBDAs in Finland are voluntarily fortified with vitamin D_2_. Similar vitamin D intake observed across diet groups in children aligns with findings from our MIRA Helsinki pilot study [20], but contrasts with earlier studies reporting lower vitamin D intake among VGN groups [22, 40–43]. Overall, vitamin D intake in our study was higher than reported in other countries due to frequent consumption of fortified dairy and PBDA products and fat spreads [21, 22].

Milk and dairy were the main sources of iodine for children in the OMN and VGT groups. In contrast, for adults—who consumed less dairy—the main sources were the ‘spices and condiments’ group, and cereals. The Finnish National Nutrition Council recommends the use of iodized salt (iodine content of 25 µg/g) in households, food industry and food services [44], and iodine supplementation is advised for those following dairy-free diets [1]. Across all diet groups, the use of iodized salt in mixed dishes (as reflected in the ‘spices and condiments’ group) and by the food industry and bakeries (as reflected in the ‘cereals’ group) formed the basis of iodine intake. However, intake was lowest among children and adults in the VGN group. Our results suggest that broader implementation of iodine fortification in PBDAs warrants consideration to address lower intakes among those following VGN diets.

### Riboflavin, vitamin B12, folate, and iron

Milk and dairy products are naturally rich in several nutrients, including riboflavin and vitamin B12 [37]. In our study, fortified PBDAs emerged as a significant dietary source of these nutrients for VGT, and particularly VGN, participants. VGT and VGN participants exhibited lower intakes of vitamin B12 compared to their OMN peers. However, vitamin B12 fortification has likely increased over the past decade, as the intake among VGN Finnish adults measured in 2011 was lower than in the present study [45].

As in prior studies, VGN diet provided the highest folate and iron intake [20, 45–47]. Notably, folate intake in OMN group was particularly low, aligning with findings from the FinDiet 2017 study, which reported inadequate folate intake in nearly 40% of women and 30% of men [13]. Moreover, evidence remains inconclusive regarding whether the lower bioavailability of iron from plant sources contributes to suboptimal iron status or, conversely, is associated with certain health benefits [48].

### Strengths and limitations

Our dietary assessment methodology has several strengths. First, we employed the National Food Composition Database (Fineli®) and carried out extensive work to compile information on new recipes and food items. Second, collaboration with municipal food services enabled the inclusion of detailed information on recipes used in ECEC centers during the study. Third, we used the GIFT tool to standardize food group classification, enhancing the comparability and alignment of our findings with future research.

Our study does face certain limitations, such as the inherent challenges of dietary intake assessment methods related to accurate reporting by participants and ECEC staff. We mitigated these by using food picture books to improve the accuracy of portion size estimation and by collecting dietary data across two weekdays and one weekend day to capture day-to-day variability. Additionally, evaluating dietary intake remains challenging due to the frequent introduction of new food products and the laborious process of updating food composition databases. Finally, the families were recruited from the Helsinki capital area, and based on the caregivers’ educational levels, likely represented higher-than-average socioeconomic backgrounds, which may limit the generalizability of our results. However, individuals following a VGN diet are predominantly concentrated in larger cities, including Helsinki (personal communication, M. Simojoki, October 2025, 10.1093/eurpub/ckaf161.1366); therefore, our results likely provide a good overview of the VGN diet in Finland.

## Conclusions

PBDs are characterized by high consumption of foods that are linked to beneficial health effects, including pulses, seeds, and nuts, which is reflected in higher intakes of dietary fiber, folate, and PUFAs compared to OMN diets. Additionally, food fortification has been instrumental in bridging gaps between OMN and PBDs, promoting comparable intakes of several key nutrients such as vitamin D, riboflavin, vitamin B12, and calcium, particularly through fortified PBDAs. However, continued efforts are required to support individuals following VGN and VGT diets, as the exclusion or reduction of animal-source foods, if not accompanied by appropriate dietary knowledge about key plant-based sources of critical nutrients, may increase the risk of inadequate intakes. Of particular concern are iodine and long-chain n-3 fatty acids.

## Supplementary Information

Below is the link to the electronic supplementary material.

Supplementary Appendix (PDF 111 kb)

## Data Availability

The datasets analyzed in this study are not publicly available due to data protection and the risk of participant identification. Data can be made available upon request and following a data-sharing agreement by contacting the MIRA2 Principal Investigator, Dr. Liisa Korkalo.

## Abbreviations

BMI: Body-mass index
DHA: Docosahexaenoic acid
ECEC: Early childhood education and care
EPA: Eicosapentaenoic acid
MUFA: Monounsaturated fatty acids
NNR: Nordic nutrition recommendation
OMN: Omnivorous diet
PBD: Plant-based diet
PBDA: Plant-based dairy alternative
PUFA: Polyunsaturated fatty acids
RI: Recommended intake
SFA: Saturated fatty acids
VGN: Vegan diet
VGT: Vegetarian diet
