# Supplementary material for: Food sources of macro- and micronutrients in young children and adults following vegan, vegetarian, and omnivorous diets
Source: Eur J Nutr. 2026 Mar 9;65(3):88. doi: 10.1007/s00394-026-03898-9 (PMC12971779; doi:10.1007/s00394-026-03898-9)
Supplement: Supplementary file 1 — Supplementary Appendix (PDF 111 kb) [file 394_2026_3898_MOESM1_ESM.pdf]

Food Sources of Macro- and Micronutrients in Young Children and Adults Following Vegan, Vegetarian, and Omnivorous Diets

Supplementary Appendix

Venla Tilli[1], Topi Hovinen[2], Elina Kettunen[1], Riitta Freese[1], Suvi T. Itkonen[1], Maijaliisa Erkkola[1], Anu Suomalainen[2-4], Liisa Korkalo[1]

[1]Department of Food and Nutrition, University of Helsinki, , Helsinki, Finland
[2]Stem Cells and Metabolism Program, Faculty of Medicine, University of Helsinki, Helsinki, Finland
[3]HiLife, University of Helsinki, Helsinki, Finland
[4] HUS Diagnostics Centre, Helsinki University Hospital, Helsinki, Finland

Corresponding author
Venla Tilli, venla.tilli@helsinki.fi

Table of contents

S1 Food groups . . . 3
S2 Food group consumption . . . 4
S3 Nutrient intakes . . . 5
S4 Energy . . . 6
S5 Protein . . . 7
S6 Carbohydrates . . . 8
S7 Fiber . . . 9
S8 Fat . . . 10
S9 Saturated fatty acids . . . 11
S10 Monounsaturated fatty acids . . . 12
S11 Polyunsaturated fatty acids . . . 13
S12 Thiamine . . . 14
S13 Riboflavin . . . 15
S14 Niacin . . . 16
S15 Vitamin B6 . . . 17
S16 Folate . . . 18
S17 Vitamin B12 . . . 19
S18 Vitamin A . . . 20
S19 Vitamin C . . . 21
S20 Vitamin D . . . 22
S21 Vitamin E . . . 23
S22 Vitamin K . . . 24
S23 Calcium . . . 25
S24 Iodine . . . 26
S25 Iron . . . 27
S26 Magnesium . . . 28
S27 Phosphorus . . . 29
S28 Potassium . . . 30
S29 Selenium . . . 31
S30 Zinc . . . 32
S31 Linoleic acid, LA . . . 33
S32 Alpha-linolenic acid, ALA . . . 34
S33 Eicosapentaenoic acid, EPA . . . 35
S34 Docosahexaenoic acid, DHA . . . 36

Table S1: Food groups and group descriptions

Main group	Group description
Cereals	Grains, flours, brans, flakes, bread, porridge, pasta, rice, cereal bars, muesli, popcorn, rice cakes, croutons, tortilla wraps, dough-based foods such as salty crepes and pastries
Milk and dairy products	Full fat-, low fat and fat-free milk, creams, yogurts, curds, ice cream, milk-based desserts, cheese
Plant-based dairy alternatives (PBDA)	Oat-, rye-, soy-based drinks, yogurts and creams, other nut-based drinks yogurts and creams, potato starch based imitation cheese
Pulses, seeds, nuts	Peas, beans (fresh, dried, canned), hummus, pea/bean patties and sausages, pea and soy protein based meat analogues, tofu, tempeh, nuts, seeds, tahini, peanut butter
Roots and tubers	Potatoes, French fries, potato salad, mashed potatoes (also from powder), sweet potatoes, tapioca flour
Eggs	Eggs (boiled, fried)
Fish	Fish fillets, smoked and canned fish, fish fingers, patties and balls, shrimps and other sea food
Meat	Red meat and poultry; bacon, liver, ground beef, cold cuts, fillets, sausages, meat balls, kebab, nuggets, wings, meat lasagna
Insects *	
Vegetables	Green leafy vegetables, fresh and cooked vegetables, carrots, beetroots, ginger, mushrooms, canned and preserved vegetables, tomato sauce, seaweed, sun-dried tomatoes, salsa, coleslaw, olives, sauerkraut
Fruits	Fresh and dried berries, fresh and dried fruits, fruit bars, canned fruit, fruit purees
Fats and oils	Vegetable oils, margarine, butter, fat mixtures (spreads)
Sweets and sugars	Sweet pastries, cookies, croissants, pies, chocolate, fruit jams and marmalade, sweets, sugar, honey, syrups
Spices and condiments	Salt, broth, dried herbs, ketchup, mustard, mayo, salad dressings, pesto, barbeque sauce, curry paste, vinegar
Beverages	Water, mineral water, sweetened mineral waters, coffee, tea, cocoa, energy drinks, soft drinks, fruit juices, nectars, spirits, wine, beer, ciders
Other	Foods for particular nutritional uses (Baby foods (porridges), protein powders, protein bars, protein shakes); Nutritional yeast, vegemite, psyllium; Food additives (Sweeteners, lifting agents, baking yeast)
Composite dishes *	
Savory snacks	Crisps

* No foods in this group were present in our data.

Table S2: Food group consumption in children and adults by diet groups

	Children				Adults			
	OMN	VGT	VGN		OMN	VGT	VGN	
Food group, g/d	n=26	n=18	n=19	p value	n=32	n=27	n=34	p value
Cereals	124	134	141	0.226	193	225	214	0.125
	[105-149]	[124-160]	[120-171]		[140-238]	[168-310]	[171-259]	
Roots, tubers	36.3	43.8	37.9	0.840	53	30.2	60.7	0.323
	[18.1-73]	[19.1-71.9]	[19.4-60.7]		[22.4-97.9]	[8.19-91.5]	[32-81.4]	
Pulses, seeds, nuts	6.78 ^{a}	48.1 ^{b}	63.1 ^{b}	<0.001	20.5 ^{a}	134 ^{b}	155 ^{b}	<0.001
	[0.835-15.6]	[27.5-69.9]	[47.5-74.5]		[4.04-54]	[65.2-172]	[128-185]	
Plant-based dairy alternatives	0 ^{a}	235 ^{b}	510 ^{c}	<0.001	15.2 ^{a}	223 ^{b}	360 ^{c}	<0.001
(PBDA)	[0-37]	[118-361]	[341-662]		[0-88.5]	[167-303]	[239-563]	
Milk, dairy products*	388	219	0	0.081	294 ^{a}	91.3 ^{b}	0	<0.001
	[237-571]	[93.4-362]	[0-0]		[126-430]	[43-127]	[0-0]	
Sum of milk, dairy, PBDA	419	479	510	0.501	306	329	360	0.503
	[291-573]	[363-625]	[341-662]		[216-470]	[225-418]	[239-563]	
Eggs*	10.3	2.68	0	0.074	14.5	3.71	0	0.378
	[2.99-19.5]	[0-7.69]	[0-0]		[4.14-27.5]	[0-36.7]	[0-0]	
Fish*	19	2.5	0	0.137	33.3 ^{a}	0 ^{b}	0	0.004
	[0-36.9]	[0-21.2]	[0-0]		[6.5-68.3]	[0-18.2]	[0-0]	
Meat*	41.8 ^{a}	0 ^{b}	0	<0.001	104 ^{a}	0 ^{b}	0	<0.001
	[24.7-71.3]	[0-0]	[0-0]		[64.7-144]	[0-0]	[0-0]	
Vegetables	103	110	89.8	0.205	193 ^{a}	343 ^{b}	223 ^{a,b}	0.003
	[64.2-139]	[104-184]	[72.3-128]		[123-264]	[222-437]	[168-325]	
Fruits	107 ^{a}	178 ^{b}	161 ^{b}	0.006	115	128	184	0.044
	[72.9-166]	[123-210]	[112-200]		[46.8-160]	[78.9-205]	[92.2-313]	
Sum of fruits, vegetables	205 ^{a}	324 ^{b}	272 ^{c}	0.001	325 ^{a}	500 ^{b}	456 ^{b}	0.002
	[159-257]	[260-414]	[214-317]		[213-412]	[352-586]	[329-574]	
Fats, oils	16.4	18.9	22.1	0.285	24.4	23.9	35.5	0.424
	[11.2-21.3]	[12.3-25.3]	[15.5-27.6]		[16.5-34]	[16.2-36]	[16.6-45.4]	
Sweets, sugars	13.4	29.4	22	0.362	36.2	33.7	26.8	0.783
	[6.82-32.2]	[11-36.4]	[10.3-34.8]		[12.9-59.1]	[17.5-56.2]	[19-50.5]	
Spices, condiments	4.73	7.45	6.82	0.232	15.8	18.1	23.3	0.537
	[1.87-8]	[4.84-8.97]	[4.01-12.5]		[7.26-27.2]	[11-32]	[7.53-33.1]	
Beverages	167	226	280	0.051	887	846	816	0.811
	[107-268]	[163-276]	[159-322]		[683-1090]	[631-1060]	[523-1140]	
Savory snacks	0	0	0	0.343	0 ^{a}	0 ^{b}	0 ^{a,b}	0.025
	[0-0]	[0-0]	[0-0]		[0-0]	[0-15]	[0-13]	
Other	0.469	0.989	1.74	0.058	1.23	0.913	1.84	0.057
	[0.0233-1.73]	[0.17-3.36]	[0.715-3.29]		[0.374-2.46]	[0.16-2.82]	[1.31-4.46]	

OMN, omnivorous diet; VGT, vegetarian diet; VGN, vegan diet.

Data are presented as medians and interquartile ranges [25th–75th percentiles].

Group differences were assessed using the Kruskal–Wallis test. For variables with $p < 0.05$, pairwise comparisons were performed using the Wilcoxon rank-sum test. P-values were adjusted for multiple testing using the Benjamini–Hochberg false discovery rate (FDR) method. Statistically significant differences (adjusted $p < 0.05$) are indicated by different superscript letters (a, b, c), i.e. medians not sharing a common superscript letter are significantly different from each other.

* For these food groups, only pairwise comparisons between OMN and VGT groups were assessed.

Table S3: Nutrient intakes in children and adults by diet groups

	Children				Adults			
	OMN	VGT	VGN		OMN	VGT	VGN	
Nutrient	n=26	n=18	n=19	p value	n=32	n=27	n=34	p value
Thiamine (vitamin B1), mg/d	0.79 ^{a}	0.97 ^{b}	1.05 ^{b}	<0.001	1.17 ^{a}	1.25 ^{a}	1.69 ^{b}	<0.001
	[0.63–0.91]	[0.87–1.18]	[0.97–1.22]		[1.0–1.45]	[1.09–1.62]	[1.33–2.03]	
mg/MJ	0.16 ^{a}	0.16 ^{a}	0.19 ^{b}	<0.001	0.15 ^{a}	0.16 ^{a}	0.20 ^{b}	<0.001
	[0.14–0.18]	[0.15–0.19]	[0.18–0.21]		[0.13–0.16]	[0.14–0.18]	[0.17–0.22]	
Niacin (vitamin B3), NE, mg/d	7.7	6.7	7.0	0.679	17.1 ^{a}	13.9 ^{b}	14.1 ^{b}	0.018
	[6.1–9.2]	[6.1–8.2]	[5.7–8.8]		[15.4–23.8]	[10.4–18.1]	[11.3–17.6]	
mg/MJ	1.5 ^{a}	1.2 ^{b}	1.2 ^{b}	0.010	2.3 ^{a}	1.7 ^{b}	1.6 ^{b}	0.005
	[1.3–2.0]	[1.1–1.3]	[1.1–1.6]		[1.7–2.7]	[1.4–1.9]	[1.3–1.9]	
Vitamin B6, mg/d	1.1 ^{a}	1.3 ^{b}	1.1 ^{a,b}	0.043	1.9	1.8	2.0	0.406
	[1.0–1.2]	[1.1–1.4]	[0.9–1.3]		[1.4–2.2]	[1.5–2.2]	[1.6–2.4]	
mg/MJ	0.22	0.21	0.20	0.451	0.22	0.20	0.22	0.339
	[0.20–0.26]	[0.19–0.27]	[0.17–0.27]		[0.19–0.25]	[0.18–0.24]	[0.19–0.29]	
Vitamin A, RAE, µg/d	528	498	449	0.681	670	825	662	0.192
	[406–647]	[429–655]	[292–679]		[482–895]	[580–1050]	[471–834]	
µg/MJ	102	98	82	0.184	78	88	72	0.151
	[82–132]	[69–104]	[57–114]		[60–102]	[78–128]	[55–111]	
Vitamin C, mg/d	72	87	72	0.145	98 ^{a}	174 ^{b}	130 ^{b}	0.001
	[51–98]	[70–109]	[52–89]		[60–135]	[126–215]	[106–169]	
mg/MJ	14	15	13	0.251	12 ^{a}	18 ^{b}	16 ^{b}	0.004
	[10–18]	[13–18]	[10–15]		[8–16]	[14–24]	[13–22]	
Vitamin E, mg/d	6.1 ^{a}	8.4 ^{b}	9.2 ^{b}	<0.001	11.1 ^{a}	14.0 ^{b}	16.3 ^{b}	0.001
	[5.0–7.4]	[7.5–10.6]	[7.4–11.6]		[9.8–12.7]	[11.9–16.5]	[11.9–21.2]	
mg/MJ	1.2 ^{a}	1.5 ^{b}	1.6 ^{b}	<0.001	1.4 ^{a}	1.7 ^{b}	1.8 ^{b}	<0.001
	[1.1–1.6]	[1.3–1.7]	[1.5–2.0]		[1.1–1.4]	[1.5–1.9]	[1.6–2.0]	
Vitamin K, µg/d	58	70	78	0.118	117 ^{a}	156 ^{a,b}	172 ^{b}	0.005
	[47–82]	[53–88]	[61–90]		[86–154]	[105–229]	[138–234]	
µg/MJ	12.6	12.4	13.5	0.216	13.6 ^{a}	20.8 ^{a,b}	20.0 ^{b}	0.003
	[10.1–14.8]	[9.78–14.4]	[11.7–17.0]		[10.3–17.3]	[11.8–24.6]	[16.1–26.3]	
Magnesium, mg/d	216 ^{a}	298 ^{b}	330 ^{b}	<0.001	389 ^{a}	476 ^{b}	550 ^{b}	<0.001
	[182–265]	[263–340]	[279–371]		[287–439]	[383–587]	[432–624]	
mg/MJ	45 ^{a}	50 ^{b}	58 ^{c}	<0.001	44 ^{a}	53 ^{b}	61 ^{c}	<0.001
	[40–52]	[46–58]	[55–63]		[40–51]	[49–62]	[55–69]	
Phosphorus, mg/d	943 ^{a,b}	988 ^{a}	840 ^{b}	0.007	1520	1420	1370	0.238
	[847–1210]	[902–1130]	[697–956]		[1130–1850]	[1180–1720]	[1100–1550]	
mg/MJ	202 ^{a}	176 ^{a}	147 ^{b}	<0.001	176 ^{a}	168 ^{a,b}	149 ^{b}	0.003
	[165–229]	[170–182]	[139–161]		[163–201]	[150–186]	[138–171]	
Potassium, mg/d	2160	2410	2170	0.162	3420	3570	3860	0.110
	[1800–2570]	[2150–2800]	[1890–2470]		[2640–4060]	[3110–4340]	[3360–4370]	
mg/MJ	437	419	381	0.052	412	421	444	0.084
	[374–483]	[380–452]	[351–436]		[355–443]	[373–474]	[392–483]	
Selenium, µg/d	40.0 ^{a}	31.7 ^{b}	21.9 ^{c}	<0.001	75.7 ^{a}	44.3 ^{b}	36.4 ^{c}	<0.001
	[36.6–48.9]	[28.3–36.5]	[19.8–26.8]		[54.9–102]	[39.5–57.4]	[27.8–48.5]	
µg/MJ	8.6 ^{a}	5.8 ^{b}	4.0 ^{c}	<0.001	9.2 ^{a}	5.4 ^{b}	4.2 ^{c}	<0.001
	[7.4–9.6]	[4.8–6.8]	[3.7–4.6]		[7.9–11.3]	[4.9–6.4]	[3.3–5.2]	
Zinc, mg/d	7.5	7.3	6.6	0.221	10.8	9.7	10.3	0.509
	[5.8–8.5]	[6.6–8.2]	[5.7–7.6]		[8.8–13.5]	[8.5–12.8]	[8.5–12.7]	
mg/MJ	1.5 ^{a}	1.3 ^{a,b}	1.2 ^{b}	0.001	1.4	1.2	1.1	0.048
	[1.2–1.6]	[1.2–1.4]	[1.0–1.3]		[1.2–1.5]	[1.1–1.4]	[1.0–1.3]	

OMN, omnivorous diet; VGT, vegetarian diet; VGN, vegan diet; NE, niacin equivalent; RAE, retinol equivalent.

Data are presented as medians and interquartile ranges [25th–75th percentiles].

Group differences were assessed using the Kruskal–Wallis test. For variables with $p < 0.05$, pairwise comparisons were performed using the Wilcoxon rank-sum test. P-values were adjusted for multiple testing using the Benjamini–Hochberg false discovery rate (FDR) method. Statistically significant differences (adjusted $p < 0.05$) are indicated by different superscript letters (a, b, c), i.e. medians not sharing a common superscript letter are significantly different from each other.

Table S4: Mean energy (kJ) intake and percentages of total energy intake by food groups in children and adults

	Children						Adults					
	OMN n=26		VGT n=18		VGN n=29		OMN n=32		VGT n=27		VGN n=34	
Food group	Mean	%	Mean	%	Mean	%	Mean	%	Mean	%	Mean	%
Cereals	1568.1	31.1	1645.2	28.5	1807.7	32.2	2219.4	25.9	2470.0	28.2	2391.1	27.5
Roots, tubers	180.3	3.6	168.5	2.9	176.3	3.1	272.7	3.2	190.0	2.2	253.5	2.9
Pulses, seeds, nuts	123.7	2.5	514.6	8.9	627.7	11.2	433.0	5.1	1137.5	13.0	1488.7	17.1
Plant-based dairy alternatives	109.9	2.2	614.4	10.7	1162.3	20.7	219.3	2.6	640.5	7.3	1262.7	14.5
Milk, dairy products	936.7	18.6	732.0	12.7	0.0	0.0	1338.2	15.6	845.8	9.7	0.0	0.0
Eggs	76.5	1.5	48.7	0.8	0.0	0.0	180.2	2.1	118.1	1.3	0.2	0.0
Fish	173.2	3.4	60.6	1.1	0.0	0.0	301.6	3.5	114.6	1.3	0.0	0.0
Meat	397.1	7.9	35.8	0.6	0.0	0.0	909.4	10.6	21.3	0.2	0.0	0.0
Vegetables	110.7	2.2	212.4	3.7	135.8	2.4	224.6	2.6	468.0	5.3	359.4	4.1
Fruits	338.8	6.7	557.2	9.7	468.2	8.3	383.2	4.5	471.0	5.4	647.6	7.4
Fats, oils	495.2	9.8	551.1	9.6	618.2	11.0	809.2	9.4	887.6	10.1	1022.7	11.7
Sweets, sugars	311.0	6.2	419.5	7.3	377.7	6.7	693.6	8.1	649.4	7.4	574.0	6.6
Spices, condiments	35.7	0.7	41.3	0.7	35.4	0.6	119.4	1.4	162.7	1.9	107.3	1.2
Beverages	122.3	2.4	84.9	1.5	153.2	2.7	370.3	4.3	363.7	4.2	370.7	4.3
Savory snacks	26.5	0.5	17.4	0.3	11.0	0.2	29.1	0.3	179.2	2.0	153.1	1.8
Other	35.8	0.7	62.6	1.1	41.2	0.7	62.1	0.7	30.3	0.3	76.7	0.9
Total	5041.5	100.0	5766.1	100.0	5614.6	100.0	8565.2	100.0	8749.8	100.0	8707.5	100.0

OMN, omnivorous diet; VGT, vegetarian diet; VGN, vegan diet.

Table S5: Mean daily protein (g) intake and percentages of total intake by food groups in children and adults

	Children						Adults					
	OMN n=26		VGT n=18		VGN n=29		OMN n=32		VGT n=27		VGN n=34	
Food group	Mean	%	Mean	%	Mean	%	Mean	%	Mean	%	Mean	%
Cereals	11.5	23.8	12.8	26.4	13.9	35.2	17.3	19.8	20.6	28.3	21.2	32.1
Roots, tubers	1.0	2.1	0.9	1.8	0.9	2.3	1.4	1.7	1.0	1.4	1.4	2.2
Pulses, seeds, nuts	1.7	3.5	8.8	18.1	9.9	25.0	4.6	5.3	19.3	26.5	22.9	34.6
Plant-based dairy alternatives	0.5	1.0	4.4	9.0	10.3	26.2	0.9	1.0	3.7	5.1	9.5	14.3
Milk, dairy products	15.3	31.7	11.2	23.1	0.0	0.0	19.5	22.4	10.6	14.6	0.0	0.0
Eggs	1.7	3.6	1.1	2.3	0.0	0.0	3.8	4.3	2.5	3.5	0.0	0.0
Fish	3.8	7.8	2.3	4.7	0.0	0.0	8.0	9.1	3.4	4.7	0.0	0.0
Meat	9.1	18.8	0.8	1.7	0.0	0.0	22.9	26.3	0.4	0.6	0.0	0.0
Vegetables	0.9	1.8	1.6	3.3	1.2	3.0	2.0	2.3	4.2	5.8	3.5	5.3
Fruits	0.9	1.9	1.6	3.4	1.2	3.1	1.1	1.3	1.4	1.9	1.9	2.8
Fats, oils	0.0	0.1	0.0	0.1	0.0	0.1	0.0	0.1	0.1	0.1	0.0	0.0
Sweets, sugars	0.7	1.4	1.3	2.6	0.8	1.9	1.9	2.1	1.4	2.0	1.1	1.6
Spices, condiments	0.1	0.3	0.2	0.3	0.2	0.5	0.4	0.4	0.5	0.8	0.4	0.6
Beverages	0.2	0.5	0.2	0.5	0.2	0.5	1.7	2.0	1.7	2.3	1.8	2.6
Savory snacks	0.1	0.2	0.1	0.1	0.0	0.1	0.1	0.1	0.6	0.8	0.5	0.7
Other	0.7	1.5	1.2	2.5	0.8	2.0	1.7	1.9	1.2	1.7	2.0	3.0
Total	48.3	100.0	48.5	100.0	39.4	100.0	87.3	100.0	72.8	100.0	66.1	100.0

OMN, omnivorous diet; VGT, vegetarian diet; VGN, vegan diet.

Table S6: Mean daily intake of carbohydrate (g) and percentages of the total intake by food groups in children and adults.

	Children						Adults					
	OMN n=26		VGT n=18		VGN n=29		OMN n=32		VGT n=27		VGN n=34	
Food group	Mean	%	Mean	%	Mean	%	Mean	%	Mean	%	Mean	%
Cereals	66.1	45.2	68.7	40.8	75.2	43.7	94.6	47.2	102.6	47.0	96.3	42.2
Roots, tubers	8.8	6.0	8.1	4.8	8.0	4.7	12.4	6.2	9.0	4.1	12.0	5.3
Pulses, seeds, nuts	1.6	1.1	7.2	4.3	7.0	4.1	5.1	2.5	13.1	6.0	17.9	7.9
Plant-based dairy alternatives	3.5	2.4	15.7	9.3	29.7	17.3	4.3	2.2	14.7	6.7	25.2	11.0
Milk, dairy products	19.7	13.5	12.6	7.5	0.0	0.0	14.6	7.3	4.9	2.2	0.0	0.0
Eggs	0.0	0.0	0.0	0.0	0.0	0.0	0.1	0.0	0.1	0.0	0.0	0.0
Fish	1.8	1.2	0.0	0.0	0.0	0.0	1.2	0.6	0.2	0.1	0.0	0.0
Meat	1.4	1.0	0.0	0.0	0.0	0.0	2.0	1.0	0.0	0.0	0.0	0.0
Vegetables	4.0	2.7	6.2	3.7	4.5	2.6	7.1	3.6	13.6	6.2	10.0	4.4
Fruits	15.3	10.5	24.9	14.8	21.1	12.2	15.2	7.6	16.0	7.3	25.5	11.2
Fats, oils	0.1	0.1	0.1	0.0	0.1	0.1	0.1	0.1	0.1	0.0	0.2	0.1
Sweets, sugars	12.7	8.7	17.1	10.2	15.6	9.1	25.4	12.7	24.9	11.4	22.5	9.8
Spices, condiments	0.9	0.6	0.9	0.6	1.2	0.7	1.2	0.6	3.1	1.4	1.7	0.7
Beverages	6.6	4.5	4.4	2.6	8.4	4.9	14.9	7.5	10.8	5.0	12.4	5.4
Savory snacks	0.7	0.5	0.6	0.4	0.3	0.2	0.7	0.4	4.8	2.2	3.6	1.6
Other	2.9	2.0	1.6	1.0	1.0	0.6	1.3	0.7	0.3	0.1	1.0	0.5
Total	146.2	100.0	168.3	100.0	172.2	100.0	200.3	100.0	218.3	100.0	228.4	100.0

OMN, omnivorous diet; VGT, vegetarian diet; VGN, vegan diet.

Table S7: Mean daily fiber (g) intake and percentages of total intake by food groups in children and adults

	Children						Adults					
	OMN n=26		VGT n=18		VGN n=29		OMN n=32		VGT n=27		VGN n=34	
Food group	Mean	%	Mean	%	Mean	%	Mean	%	Mean	%	Mean	%
Cereals	8.0	50.4	9.5	39.6	9.6	37.0	10.1	45.9	13.0	37.7	12.6	32.9
Roots, tubers	0.6	3.7	0.5	2.2	0.5	2.0	0.7	3.3	0.7	2.2	0.9	2.3
Pulses, seeds, nuts	1.0	6.0	3.0	12.7	3.5	13.3	1.9	8.5	5.9	17.3	8.2	21.4
Plant-based dairy alternatives	0.5	3.0	2.5	10.6	5.4	20.9	0.6	2.8	2.3	6.6	4.0	10.5
Milk, dairy products	0.1	0.4	0.0	0.1	0.0	0.0	0.2	0.9	0.0	0.0	0.0	0.0
Eggs	0.0	0.0	0.0	0.0	0.0	0.0	0.0	0.0	0.0	0.0	0.0	0.0
Fish	0.1	0.8	0.0	0.0	0.0	0.0	0.1	0.3	0.0	0.1	0.0	0.0
Meat	0.1	0.6	0.0	0.0	0.0	0.0	0.1	0.4	0.0	0.0	0.0	0.0
Vegetables	1.8	11.2	2.7	11.2	1.9	7.5	3.4	15.3	6.2	18.2	5.1	13.4
Fruits	2.8	17.7	4.7	19.7	3.9	15.0	3.5	16.1	4.0	11.8	5.2	13.6
Fats, oils	0.0	0.0	0.0	0.0	0.0	0.0	0.0	0.0	0.0	0.0	0.0	0.0
Sweets, sugars	0.3	1.6	0.4	1.6	0.5	1.8	0.6	2.7	1.2	3.6	0.9	2.2
Spices, condiments	0.1	0.7	0.1	0.4	0.2	0.6	0.3	1.2	0.3	0.8	0.3	0.7
Beverages	0.1	0.7	0.2	0.9	0.2	0.6	0.3	1.2	0.2	0.5	0.3	0.7
Savory snacks	0.1	0.8	0.0	0.1	0.0	0.1	0.1	0.3	0.3	0.9	0.3	0.8
Other	0.4	2.2	0.3	1.1	0.3	1.1	0.2	1.1	0.2	0.5	0.6	1.4
Total	15.9	100.0	24.0	100.0	25.9	100.0	22.0	100.0	34.4	100.0	38.3	100.0

OMN, omnivorous diet; VGT, vegetarian diet; VGN, vegan diet.

Table S8: Mean daily fat (g) intake and percentages of total intake by food groups in children and adults

	Children						Adults					
	OMN n=26		VGT n=18		VGN n=29		OMN n=32		VGT n=27		VGN n=34	
Food group	Mean	%	Mean	%	Mean	%	Mean	%	Mean	%	Mean	%
Cereals	5.1	11.7	5.2	10.3	6.0	12.2	6.5	7.1	7.6	8.3	8.0	9.1
Roots, tubers	0.1	0.3	0.2	0.5	0.5	1.0	0.7	0.8	0.3	0.3	0.4	0.4
Pulses, seeds, nuts	1.6	3.6	6.0	11.8	8.4	17.3	6.9	7.6	14.7	16.1	19.7	22.3
Plant-based dairy alternatives	1.2	2.6	6.9	13.7	12.4	25.3	3.4	3.8	8.5	9.3	17.6	19.9
Milk, dairy products	8.9	20.1	8.6	17.0	0.0	0.0	20.1	22.0	15.6	17.2	0.0	0.0
Eggs	1.3	2.8	0.8	1.5	0.0	0.0	3.1	3.4	2.0	2.2	0.0	0.0
Fish	2.1	4.8	0.6	1.2	0.0	0.0	3.9	4.3	1.4	1.5	0.0	0.0
Meat	5.8	13.3	0.6	1.2	0.0	0.0	12.9	14.1	0.4	0.4	0.0	0.0
Vegetables	0.3	0.6	1.5	2.9	0.5	1.1	1.0	1.1	2.8	3.1	2.2	2.5
Fruits	0.8	1.7	1.4	2.7	1.2	2.4	1.7	1.8	3.6	3.9	3.3	3.7
Fats, oils	13.3	30.2	14.8	29.4	16.7	34.0	21.8	23.8	23.9	26.3	27.5	31.3
Sweets, sugars	2.2	4.9	2.8	5.5	2.5	5.2	6.1	6.6	4.7	5.2	4.4	5.0
Spices, condiments	0.4	1.0	0.6	1.1	0.2	0.5	2.4	2.6	2.6	2.9	1.8	2.1
Beverages	0.1	0.2	0.1	0.2	0.1	0.2	0.3	0.3	0.5	0.5	0.5	0.5
Savory snacks	0.3	0.7	0.1	0.3	0.2	0.3	0.4	0.4	2.2	2.5	2.2	2.5
Other	0.6	1.3	0.3	0.7	0.2	0.5	0.3	0.3	0.1	0.1	0.6	0.7
Total	44.0	100.0	50.4	100.0	48.9	100.0	91.6	100.0	90.9	100.0	88.1	100.0

OMN, omnivorous diet; VGT, vegetarian diet; VGN, vegan diet.

Table S9: Mean daily saturated fatty acid (g) intake and percentages of total intake by food groups in children and adults

	Children						Adults					
	OMN n=26		VGT n=18		VGN n=29		OMN n=32		VGT n=27		VGN n=34	
Food group	Mean	%	Mean	%	Mean	%	Mean	%	Mean	%	Mean	%
Cereals	0.8	5.2	0.9	6.0	1.0	9.4	1.2	3.6	1.4	4.9	1.4	7.2
Roots, tubers	0.0	0.3	0.1	0.5	0.1	0.8	0.2	0.5	0.1	0.3	0.1	0.5
Pulses, seeds, nuts	0.3	1.7	1.0	6.2	1.2	11.4	1.1	3.5	2.3	8.2	2.9	14.6
Plant-based dairy alternatives	0.3	2.0	2.5	16.3	3.2	30.5	1.4	4.3	3.2	11.5	6.5	32.5
Milk, dairy products	5.5	36.8	5.1	33.0	0.0	0.0	13.0	40.3	9.8	35.6	0.0	0.0
Eggs	0.3	2.2	0.2	1.3	0.0	0.0	0.8	2.4	0.5	1.8	0.0	0.0
Fish	0.3	1.9	0.1	0.7	0.0	0.0	0.7	2.1	0.3	0.9	0.0	0.0
Meat	2.2	14.4	0.3	1.7	0.0	0.0	4.6	14.4	0.2	0.6	0.0	0.0
Vegetables	0.0	0.2	0.2	1.0	0.1	0.6	0.1	0.4	0.4	1.5	0.3	1.3
Fruits	0.1	0.6	0.2	1.1	0.1	1.4	0.2	0.6	0.5	1.8	0.4	2.2
Fats, oils	3.8	25.5	3.4	22.1	3.4	32.8	5.4	16.7	5.9	21.4	5.4	26.8
Sweets, sugars	1.1	7.3	1.3	8.3	1.2	11.7	3.1	9.5	2.5	9.1	2.2	10.8
Spices, condiments	0.1	0.4	0.1	0.5	0.1	0.6	0.2	0.7	0.3	1.1	0.2	1.1
Beverages	0.0	0.2	0.1	0.4	0.0	0.4	0.1	0.3	0.1	0.3	0.1	0.4
Savory snacks	0.0	0.3	0.0	0.1	0.0	0.2	0.0	0.2	0.3	1.0	0.3	1.4
Other	0.2	1.0	0.1	0.8	0.0	0.3	0.2	0.5	0.0	0.1	0.2	1.1
Total	15.0	100.0	15.3	100.0	10.4	100.0	32.3	100.0	27.6	100.0	20.0	100.0

OMN, omnivorous diet; VGT, vegetarian diet; VGN, vegan diet.

Table S10: Mean daily monounsaturated fatty acid (g) intake and percentages of total intake by food groups in children and adults

	Children						Adults					
	OMN n=26		VGT n=18		VGN n=29		OMN n=32		VGT n=27		VGN n=34	
Food group	Mean	%	Mean	%	Mean	%	Mean	%	Mean	%	Mean	%
Cereals	1.9	11.7	1.8	9.7	2.1	11.2	2.2	6.3	2.6	7.6	2.8	7.9
Roots, tubers	0.0	0.2	0.1	0.3	0.2	1.2	0.3	0.7	0.1	0.2	0.1	0.4
Pulses, seeds, nuts	0.6	3.6	2.3	12.2	3.3	17.2	3.0	8.7	5.2	15.1	7.2	20.5
Plant-based dairy alternatives	0.5	2.7	2.0	10.6	3.6	18.9	1.1	3.3	2.6	7.6	4.9	13.8
Milk, dairy products	2.3	14.1	2.5	13.1	0.0	0.0	4.9	14.1	3.9	11.4	0.0	0.0
Eggs	0.5	2.9	0.3	1.6	0.0	0.0	1.2	3.5	0.8	2.3	0.0	0.0
Fish	0.9	5.5	0.2	1.0	0.0	0.0	1.4	4.2	0.6	1.6	0.0	0.0
Meat	2.6	15.5	0.2	1.1	0.0	0.0	5.7	16.3	0.1	0.4	0.0	0.0
Vegetables	0.0	0.3	0.7	3.6	0.2	1.0	0.4	1.0	1.3	3.7	1.0	2.9
Fruits	0.3	1.7	0.5	2.5	0.5	2.5	0.8	2.3	2.1	6.0	1.7	5.0
Fats, oils	5.8	34.8	6.9	36.8	8.1	42.3	10.5	30.2	11.4	33.0	13.8	39.3
Sweets, sugars	0.7	4.0	0.9	4.9	0.8	4.2	1.9	5.3	1.5	4.3	1.4	4.1
Spices, condiments	0.2	1.1	0.3	1.5	0.1	0.5	1.2	3.4	1.3	3.7	1.0	2.8
Beverages	0.0	0.1	0.0	0.1	0.0	0.1	0.0	0.1	0.2	0.5	0.1	0.3
Savory snacks	0.1	0.7	0.1	0.4	0.1	0.3	0.1	0.4	0.9	2.5	0.8	2.3
Other	0.2	1.1	0.1	0.5	0.1	0.5	0.1	0.2	0.0	0.1	0.2	0.7
Total	16.6	100.0	18.8	100.0	19.1	100.0	34.8	100.0	34.6	100.0	35.1	100.0

OMN, omnivorous diet; VGT, vegetarian diet; VGN, vegan diet.

Table S11: Mean daily polyunsaturated fatty acid (g) intake and percentages of total intake by food groups in children and adults

	Children						Adults					
	OMN n=26		VGT n=18		VGN n=29		OMN n=32		VGT n=27		VGN n=34	
Food group	Mean	%	Mean	%	Mean	%	Mean	%	Mean	%	Mean	%
Cereals	1.8	21.6	1.8	15.4	2.2	14.3	2.3	14.6	2.6	13.0	2.7	10.8
Roots, tubers	0.0	0.5	0.0	0.4	0.1	0.8	0.2	1.0	0.1	0.5	0.1	0.4
Pulses, seeds, nuts	0.6	6.9	2.0	17.4	3.0	19.8	2.1	13.2	5.6	27.7	7.5	29.9
Plant-based dairy alternatives	0.3	4.1	1.9	16.2	4.3	28.5	0.7	4.6	2.1	10.6	4.6	18.2
Milk, dairy products	0.3	3.9	0.4	3.7	0.0	0.0	0.5	3.4	0.5	2.4	0.0	0.0
Eggs	0.2	2.2	0.1	1.1	0.0	0.0	0.5	3.2	0.3	1.6	0.0	0.0
Fish	0.7	8.5	0.2	1.7	0.0	0.0	1.4	8.6	0.5	2.3	0.0	0.0
Meat	0.7	8.1	0.0	0.2	0.0	0.0	1.7	10.5	0.0	0.1	0.0	0.0
Vegetables	0.1	1.3	0.4	3.8	0.2	1.3	0.3	1.9	0.7	3.6	0.6	2.2
Fruits	0.1	1.4	0.2	1.8	0.2	1.1	0.2	1.4	0.4	1.8	0.4	1.5
Fats, oils	2.8	33.4	3.9	33.4	4.7	30.7	4.6	28.4	5.2	26.1	7.4	29.3
Sweets, sugars	0.2	2.4	0.3	2.6	0.4	2.3	0.4	2.7	0.3	1.3	0.5	2.0
Spices, condiments	0.1	1.7	0.2	1.4	0.0	0.3	0.8	5.1	0.9	4.5	0.5	2.0
Beverages	0.0	0.2	0.0	0.1	0.0	0.1	0.0	0.2	0.1	0.5	0.1	0.3
Savory snacks	0.1	1.5	0.1	0.4	0.1	0.4	0.2	1.0	0.8	4.1	0.8	3.1
Other	0.2	2.5	0.0	0.3	0.1	0.5	0.0	0.1	0.0	0.1	0.1	0.2
Total	8.4	100.0	11.6	100.0	15.2	100.0	16.0	100.0	20.1	100.0	25.1	100.0

OMN, omnivorous diet; VGT, vegetarian diet; VGN, vegan diet.

Table S12: Mean daily thiamine (mg) intake and percentages of total intake by food groups in children and adults

	Children						Adults					
	OMN n=26		VGT n=18		VGN n=29		OMN n=32		VGT n=27		VGN n=34	
Food group	Mean	%	Mean	%	Mean	%	Mean	%	Mean	%	Mean	%
Cereals	0.2	26.1	0.2	23.1	0.3	22.1	0.3	21.4	0.3	23.1	0.3	18.7
Roots, tubers	0.1	11.1	0.1	7.0	0.1	6.2	0.1	9.6	0.1	5.6	0.1	6.6
Pulses, seeds, nuts	0.0	5.6	0.1	13.4	0.2	15.8	0.1	7.6	0.3	21.8	0.4	23.8
Plant-based dairy alternatives	0.0	2.5	0.1	11.3	0.4	30.2	0.0	2.0	0.1	7.6	0.3	18.6
Milk, dairy products	0.1	17.4	0.1	8.4	0.0	0.0	0.1	7.7	0.0	2.2	0.0	0.0
Eggs	0.0	1.9	0.0	0.9	0.0	0.0	0.0	2.6	0.0	1.5	0.0	0.0
Fish	0.0	2.8	0.0	1.4	0.0	0.0	0.1	4.3	0.0	1.3	0.0	0.0
Meat	0.1	11.1	0.0	0.7	0.0	0.0	0.3	25.0	0.0	0.3	0.0	0.0
Vegetables	0.1	7.6	0.1	9.4	0.1	5.6	0.1	8.8	0.2	14.3	0.2	9.5
Fruits	0.1	6.7	0.1	8.5	0.1	5.8	0.1	6.4	0.1	5.6	0.1	6.1
Fats, oils	0.0	0.0	0.0	0.3	0.0	0.1	0.0	0.0	0.0	0.6	0.0	0.2
Sweets, sugars	0.0	0.7	0.0	1.1	0.0	0.4	0.0	1.2	0.0	0.8	0.0	0.7
Spices, condiments	0.0	1.0	0.0	0.5	0.0	1.1	0.0	0.5	0.0	0.5	0.0	0.6
Beverages	0.0	1.9	0.0	0.7	0.0	2.4	0.0	1.9	0.0	2.4	0.0	2.3
Savory snacks	0.0	0.5	0.0	0.2	0.0	0.1	0.0	0.2	0.0	1.1	0.0	0.8
Other	0.0	3.0	0.1	13.2	0.1	10.2	0.0	0.9	0.2	11.3	0.2	12.1
Total	0.8	100.0	1.1	100.0	1.2	100.0	1.3	100.0	1.5	100.0	1.8	100.0

OMN, omnivorous diet; VGT, vegetarian diet; VGN, vegan diet.

Table S13: Mean daily riboflavin (mg) intake and percentages of total intake by food groups in children and adults

	Children						Adults					
	OMN n=26		VGT n=18		VGN n=29		OMN n=32		VGT n=27		VGN n=34	
Food group	Mean	%	Mean	%	Mean	%	Mean	%	Mean	%	Mean	%
Cereals	0.1	8.2	0.1	8.0	0.1	8.2	0.1	7.4	0.2	9.6	0.2	10.9
Roots, tubers	0.0	1.1	0.0	0.9	0.0	0.8	0.0	1.1	0.0	1.0	0.0	1.2
Pulses, seeds, nuts	0.0	1.0	0.0	3.3	0.1	5.2	0.0	2.1	0.1	7.7	0.2	10.9
Plant-based dairy alternatives	0.1	7.7	0.4	29.6	1.0	64.8	0.1	6.5	0.4	24.4	0.7	39.4
Milk, dairy products	0.8	55.9	0.5	34.4	0.0	0.0	0.6	34.5	0.2	13.7	0.0	0.0
Eggs	0.1	3.8	0.0	2.3	0.0	0.0	0.1	6.3	0.1	4.5	0.0	0.0
Fish	0.0	1.5	0.0	1.0	0.0	0.0	0.1	3.0	0.0	1.4	0.0	0.0
Meat	0.1	5.7	0.0	0.7	0.0	0.0	0.2	12.3	0.0	0.4	0.0	0.0
Vegetables	0.1	5.6	0.1	5.9	0.1	5.1	0.1	8.0	0.3	15.2	0.2	13.5
Fruits	0.0	3.5	0.1	5.5	0.1	4.5	0.1	3.9	0.1	4.4	0.1	5.9
Fats, oils	0.0	0.1	0.0	0.3	0.0	0.1	0.0	0.1	0.0	0.8	0.0	0.3
Sweets, sugars	0.0	1.0	0.0	1.9	0.0	0.5	0.1	3.0	0.0	2.4	0.0	1.0
Spices, condiments	0.0	0.5	0.0	0.5	0.0	0.8	0.0	0.5	0.0	1.1	0.0	0.9
Beverages	0.0	0.8	0.0	0.8	0.0	0.9	0.1	8.2	0.1	5.7	0.1	5.3
Savory snacks	0.0	0.1	0.0	0.1	0.0	0.0	0.0	0.1	0.0	0.4	0.0	0.3
Other	0.0	3.3	0.1	4.8	0.1	9.1	0.1	3.0	0.1	7.4	0.2	10.5
Total	1.4	100.0	1.5	100.0	1.5	100.0	1.8	100.0	1.7	100.0	1.7	100.0

OMN, omnivorous diet; VGT, vegetarian diet; VGN, vegan diet.

Table S14: Mean daily niacin (mg) intake (in niacin equivalents, NE) and percentages of total intake by food groups in children and adults

	Children						Adults					
	OMN n=26		VGT n=18		VGN n=29		OMN n=32		VGT n=27		VGN n=34	
Food group	Mean	%	Mean	%	Mean	%	Mean	%	Mean	%	Mean	%
Cereals	1.7	20.1	1.9	24.6	2.0	26.9	2.0	10.0	3.1	19.8	2.6	17.2
Roots, tubers	0.3	3.8	0.3	3.4	0.3	3.6	0.4	2.2	0.3	2.0	0.4	2.9
Pulses, seeds, nuts	0.2	2.4	0.8	10.6	1.1	15.0	0.6	2.8	1.9	12.3	2.6	17.3
Plant-based dairy alternatives	0.1	0.8	0.3	4.3	1.1	14.1	0.1	0.5	0.3	2.2	1.3	8.3
Milk, dairy products	0.4	5.1	0.3	3.3	0.0	0.0	0.3	1.7	0.1	0.6	0.0	0.0
Eggs	0.0	0.1	0.0	0.1	0.0	0.0	0.0	0.1	0.0	0.1	0.0	0.0
Fish	1.2	14.6	0.6	7.4	0.0	0.0	2.7	13.7	1.2	7.7	0.0	0.0
Meat	2.6	31.0	0.2	2.6	0.0	0.0	6.8	34.5	0.1	0.7	0.0	0.0
Vegetables	0.7	8.2	0.9	11.9	0.7	9.6	1.4	7.2	2.7	17.6	1.9	12.8
Fruits	0.5	5.9	0.9	11.3	0.7	9.1	0.6	3.2	0.7	4.8	1.0	6.7
Fats, oils	0.0	0.0	0.0	0.0	0.0	0.0	0.0	0.0	0.0	0.0	0.0	0.0
Sweets, sugars	0.1	0.7	0.1	1.8	0.1	0.9	0.2	0.8	0.1	0.7	0.2	1.0
Spices, condiments	0.0	0.6	0.1	0.6	0.1	0.9	0.1	0.4	0.2	1.2	0.1	0.9
Beverages	0.1	0.9	0.0	0.4	0.1	1.1	4.3	21.8	2.5	16.4	2.6	17.1
Savory snacks	0.0	0.5	0.0	0.3	0.0	0.2	0.0	0.2	0.3	1.9	0.3	1.7
Other	0.4	5.2	1.4	17.4	1.4	18.6	0.2	0.9	1.9	12.0	2.2	14.2
Total	8.3	100.0	7.9	100.0	7.6	100.0	19.6	100.0	15.4	100.0	15.2	100.0

OMN, omnivorous diet; VGT, vegetarian diet; VGN, vegan diet.

Table S15: Mean daily vitamin B6 (mg) intake and percentages of total intake by food groups in children and adults

	Children						Adults					
	OMN n=26		VGT n=18		VGN n=29		OMN n=32		VGT n=27		VGN n=34	
Food group	Mean	%	Mean	%	Mean	%	Mean	%	Mean	%	Mean	%
Cereals	0.2	15.1	0.2	14.1	0.2	16.4	0.2	9.4	0.2	12.9	0.3	13.8
Roots, tubers	0.1	5.8	0.1	4.2	0.1	5.0	0.1	4.4	0.1	3.8	0.1	5.3
Pulses, seeds, nuts	0.0	2.2	0.1	7.4	0.1	9.6	0.1	3.3	0.2	12.4	0.3	14.6
Plant-based dairy alternatives	0.0	3.2	0.1	8.7	0.3	24.2	0.0	0.5	0.1	6.1	0.3	14.2
Milk, dairy products	0.2	17.8	0.1	10.0	0.0	0.0	0.2	7.3	0.0	2.6	0.0	0.0
Eggs	0.0	1.6	0.0	0.9	0.0	0.0	0.0	2.0	0.0	1.5	0.0	0.0
Fish	0.1	7.4	0.0	3.3	0.0	0.0	0.2	10.8	0.1	4.1	0.0	0.0
Meat	0.2	16.2	0.0	0.9	0.0	0.0	0.5	22.2	0.0	0.3	0.0	0.0
Vegetables	0.1	9.1	0.2	12.4	0.1	8.8	0.2	9.9	0.5	25.9	0.3	15.7
Fruits	0.2	15.7	0.4	28.1	0.3	23.8	0.2	11.5	0.3	15.4	0.4	20.3
Fats, oils	0.0	0.0	0.0	0.3	0.0	0.1	0.0	0.0	0.0	0.6	0.0	0.2
Sweets, sugars	0.0	0.6	0.0	0.9	0.0	0.5	0.0	0.9	0.0	0.6	0.0	0.5
Spices, condiments	0.0	0.6	0.0	0.5	0.0	0.9	0.0	0.5	0.0	1.1	0.0	0.6
Beverages	0.0	1.5	0.0	0.7	0.0	2.2	0.3	16.4	0.1	3.4	0.1	4.8
Savory snacks	0.0	0.6	0.0	0.3	0.0	0.2	0.0	0.3	0.0	2.3	0.0	1.9
Other	0.0	2.8	0.1	7.4	0.1	8.3	0.0	0.6	0.1	6.9	0.2	8.0
Total	1.1	100.0	1.3	100.0	1.2	100.0	2.1	100.0	1.8	100.0	2.1	100.0

OMN, omnivorous diet; VGT, vegetarian diet; VGN, vegan diet.

Table S16: Mean daily folate (µg) intake and percentages of total intake by food groups in children and adults

	Children						Adults					
	OMN n=26		VGT n=18		VGN n=29		OMN n=32		VGT n=27		VGN n=34	
Food group	Mean	%	Mean	%	Mean	%	Mean	%	Mean	%	Mean	%
Cereals	37.7	23.3	40.2	17.1	44.0	15.0	48.5	17.9	57.1	14.0	61.2	13.0
Roots, tubers	9.7	6.0	8.1	3.5	7.6	2.6	13.1	4.8	9.6	2.3	12.2	2.6
Pulses, seeds, nuts	8.8	5.4	36.7	15.6	47.9	16.4	24.5	9.1	84.4	20.6	105.7	22.4
Plant-based dairy alternatives	6.4	3.9	38.7	16.5	111.5	38.1	9.0	3.3	37.1	9.1	100.2	21.2
Milk, dairy products	22.2	13.7	15.2	6.5	0.0	0.0	26.7	9.9	11.9	2.9	0.0	0.0
Eggs	7.8	4.8	4.9	2.1	0.0	0.0	17.4	6.4	11.4	2.8	0.0	0.0
Fish	2.9	1.8	1.2	0.5	0.0	0.0	5.3	2.0	2.1	0.5	0.0	0.0
Meat	4.5	2.8	0.3	0.1	0.0	0.0	12.9	4.8	0.1	0.0	0.0	0.0
Vegetables	28.0	17.3	33.2	14.1	31.1	10.6	59.0	21.8	120.3	29.4	95.5	20.3
Fruits	14.1	8.7	29.5	12.5	18.0	6.1	19.5	7.2	21.6	5.3	32.7	6.9
Fats, oils	0.1	0.1	0.6	0.3	0.2	0.1	0.1	0.1	1.7	0.4	0.7	0.1
Sweets, sugars	1.9	1.2	4.9	2.1	2.0	0.7	4.2	1.6	2.7	0.7	3.8	0.8
Spices, condiments	0.7	0.4	1.7	0.7	1.7	0.6	1.7	0.6	3.2	0.8	3.3	0.7
Beverages	5.2	3.2	1.5	0.7	5.1	1.7	11.7	4.3	17.9	4.4	18.5	3.9
Savory snacks	0.8	0.5	0.2	0.1	0.2	0.1	0.9	0.3	2.8	0.7	3.2	0.7
Other	11.6	7.1	18.0	7.7	23.5	8.0	16.2	6.0	25.2	6.2	34.6	7.3
Total	162.3	100.0	234.9	100.0	292.7	100.0	270.7	100.0	409.0	100.0	471.6	100.0

OMN, omnivorous diet; VGT, vegetarian diet; VGN, vegan diet.

Table S17: Mean daily vitamin B12 (μg) intake and percentages of total intake by food groups in children and adults

	Children						Adults					
	OMN n=26		VGT n=18		VGN n=29		OMN n=32		VGT n=27		VGN n=34	
Food group	Mean	%	Mean	%	Mean	%	Mean	%	Mean	%	Mean	%
Cereals	0.0	0.3	0.0	0.4	0.0	0.1	0.0	0.2	0.0	0.2	0.0	0.3
Roots, tubers	0.0	0.0	0.0	0.0	0.0	0.0	0.0	0.0	0.0	0.1	0.0	0.0
Pulses, seeds, nuts	0.0	0.0	0.0	0.0	0.0	0.1	0.0	0.0	0.0	0.0	0.0	0.4
Plant-based dairy alternatives	0.2	5.5	0.9	32.0	2.1	90.6	0.2	4.2	0.9	26.4	1.7	82.9
Milk, dairy products	1.7	47.6	1.1	40.0	0.0	0.0	1.6	27.1	0.7	20.9	0.0	0.0
Eggs	0.3	9.1	0.2	7.3	0.0	0.0	0.7	12.5	0.5	13.3	0.0	0.0
Fish	0.7	21.0	0.3	11.8	0.0	0.0	1.5	26.3	1.0	29.3	0.0	0.0
Meat	0.5	14.7	0.0	1.7	0.0	0.0	1.3	23.3	0.0	0.8	0.0	0.0
Vegetables	0.0	0.0	0.0	0.2	0.0	0.0	0.0	0.0	0.0	0.2	0.0	0.5
Fruits	0.0	0.0	0.0	0.0	0.0	0.0	0.0	0.0	0.0	0.0	0.0	0.0
Fats, oils	0.0	0.0	0.0	0.3	0.0	0.1	0.0	0.0	0.0	0.6	0.0	0.4
Sweets, sugars	0.0	0.4	0.0	1.2	0.0	0.0	0.0	0.4	0.0	0.6	0.0	0.1
Spices, condiments	0.0	0.0	0.0	0.3	0.0	0.0	0.0	0.3	0.0	0.3	0.0	0.0
Beverages	0.0	0.2	0.0	0.6	0.0	0.0	0.3	4.9	0.0	1.0	0.0	0.7
Savory snacks	0.0	0.0	0.0	0.0	0.0	0.0	0.0	0.0	0.0	0.0	0.0	0.0
Other	0.0	1.1	0.1	4.2	0.2	9.0	0.1	0.9	0.2	6.3	0.3	14.6
Total	3.5	100.0	2.9	100.0	2.4	100.0	5.7	100.0	3.4	100.0	2.1	100.0

OMN, omnivorous diet; VGT, vegetarian diet; VGN, vegan diet.

Table S18: Mean daily vitamin A (µg) intake (in retinol equivalents, RAE) and percentages of total intake by food groups in children and adults

	Children						Adults					
	OMN n=26		VGT n=18		VGN n=29		OMN n=32		VGT n=27		VGN n=34	
Food group	Mean	%	Mean	%	Mean	%	Mean	%	Mean	%	Mean	%
Cereals	4.7	0.9	3.6	0.7	1.7	0.4	3.1	0.4	3.7	0.4	4.8	0.6
Roots, tubers	14.4	2.7	25.7	4.9	17.2	3.5	11.0	1.4	58.9	6.6	43.7	5.7
Pulses, seeds, nuts	3.1	0.6	7.6	1.5	6.5	1.3	3.8	0.5	11.3	1.3	14.7	1.9
Plant-based dairy alternatives	14.6	2.7	32.8	6.3	63.5	12.8	0.1	0.0	32.7	3.6	59.4	7.8
Milk, dairy products	66.6	12.5	63.8	12.3	0.0	0.0	174.2	22.8	138.2	15.4	0.0	0.0
Eggs	34.0	6.4	21.8	4.2	0.0	0.0	77.4	10.1	49.0	5.5	0.1	0.0
Fish	1.6	0.3	0.9	0.2	0.0	0.0	4.4	0.6	2.0	0.2	0.0	0.0
Meat	9.5	1.8	0.5	0.1	0.0	0.0	56.8	7.4	0.3	0.0	0.0	0.0
Vegetables	236.3	44.4	217.7	41.8	237.3	47.8	282.7	36.9	473.2	52.7	443.3	57.9
Fruits	9.7	1.8	18.4	3.5	11.6	2.3	12.4	1.6	12.0	1.3	18.9	2.5
Fats, oils	101.2	19.0	101.5	19.5	129.5	26.1	102.5	13.4	92.6	10.3	161.9	21.1
Sweets, sugars	6.1	1.1	14.8	2.8	5.5	1.1	16.7	2.2	11.6	1.3	2.6	0.3
Spices, condiments	13.8	2.6	8.4	1.6	22.3	4.5	16.8	2.2	9.2	1.0	11.2	1.5
Beverages	0.8	0.2	0.6	0.1	0.7	0.1	0.8	0.1	2.1	0.2	3.3	0.4
Savory snacks	0.7	0.1	1.2	0.2	0.0	0.0	1.4	0.2	0.4	0.0	1.7	0.2
Other	15.7	2.9	0.8	0.1	0.8	0.2	1.5	0.2	0.1	0.0	0.0	0.0
Total	532.7	100.0	520.3	100.0	496.7	100.0	765.6	100.0	897.3	100.0	765.6	100.0

OMN, omnivorous diet; VGT, vegetarian diet; VGN, vegan diet.

Table S19: Mean daily vitamin C (mg) intake and percentages of total intake by food groups in children and adults

	Children						Adults					
	OMN n=26		VGT n=18		VGN n=29		OMN n=32		VGT n=27		VGN n=34	
Food group	Mean	%	Mean	%	Mean	%	Mean	%	Mean	%	Mean	%
Cereals	1.2	1.6	0.2	0.2	0.5	0.7	0.7	0.7	0.5	0.3	1.6	0.9
Roots, tubers	4.0	5.3	3.2	3.5	3.1	3.9	5.4	5.0	3.8	2.0	5.3	3.1
Pulses, seeds, nuts	0.5	0.7	1.1	1.2	1.9	2.4	1.1	1.0	4.2	2.3	2.8	1.6
Plant-based dairy alternatives	0.0	0.0	0.8	0.9	4.6	5.9	0.1	0.1	1.0	0.5	2.1	1.2
Milk, dairy products	6.8	9.0	2.9	3.2	0.0	0.0	4.2	3.9	0.7	0.4	0.0	0.0
Eggs	0.0	0.0	0.0	0.0	0.0	0.0	0.0	0.0	0.0	0.0	0.0	0.0
Fish	0.1	0.1	0.0	0.0	0.0	0.0	0.1	0.1	0.0	0.0	0.0	0.0
Meat	0.1	0.1	0.0	0.0	0.0	0.0	0.3	0.2	0.0	0.0	0.0	0.0
Vegetables	26.7	35.4	38.4	42.0	26.8	34.2	46.5	43.8	125.8	68.4	89.0	51.9
Fruits	24.3	32.3	39.2	42.9	26.6	33.9	33.3	31.4	33.0	17.9	50.4	29.4
Fats, oils	0.0	0.0	0.0	0.0	0.0	0.0	0.0	0.0	0.0	0.0	0.0	0.0
Sweets, sugars	0.4	0.6	0.3	0.3	0.3	0.4	0.5	0.5	0.5	0.3	0.5	0.3
Spices, condiments	0.4	0.5	0.4	0.4	0.5	0.6	0.6	0.5	1.1	0.6	0.6	0.3
Beverages	8.8	11.6	4.5	4.9	13.3	16.9	13.5	12.7	13.2	7.2	18.9	11.0
Savory snacks	0.1	0.1	0.0	0.0	0.0	0.0	0.1	0.1	0.2	0.1	0.2	0.1
Other	2.1	2.8	0.5	0.6	0.8	1.0	0.1	0.1	0.0	0.0	0.0	0.0
Total	75.3	100.0	91.4	100.0	78.4	100.0	106.3	100.0	183.9	100.0	171.4	100.0

OMN, omnivorous diet; VGT, vegetarian diet; VGN, vegan diet.

Table S20: Mean daily vitamin D (µg) intake and percentages of total intake by food groups in children and adults

	Children						Adults					
	OMN n=26		VGT n=18		VGN n=29		OMN n=32		VGT n=27		VGN n=34	
Food group	Mean	%	Mean	%	Mean	%	Mean	%	Mean	%	Mean	%
Cereals	0.0	0.5	0.0	0.3	0.0	0.1	0.0	0.2	0.0	0.2	0.0	0.3
Roots, tubers	0.0	0.0	0.0	0.0	0.0	0.0	0.0	0.1	0.0	0.0	0.0	0.0
Pulses, seeds, nuts	0.0	0.0	0.0	0.0	0.0	0.0	0.0	0.0	0.0	0.0	0.0	0.1
Plant-based dairy alternatives	0.6	6.8	2.2	30.5	4.3	54.1	0.6	7.2	2.0	31.9	3.2	43.3
Milk, dairy products	2.9	35.7	1.5	21.6	0.0	0.0	1.3	14.3	0.3	4.7	0.0	0.0
Eggs	0.3	3.6	0.2	2.5	0.0	0.0	0.7	7.7	0.4	6.9	0.0	0.0
Fish	1.1	13.5	0.6	8.7	0.0	0.0	3.0	33.9	1.3	21.2	0.0	0.0
Meat	0.2	1.9	0.0	0.1	0.0	0.0	0.4	4.6	0.0	0.1	0.0	0.0
Vegetables	0.0	0.2	0.0	0.2	0.0	0.0	0.1	0.8	0.1	0.9	0.0	0.1
Fruits	0.0	0.0	0.0	0.0	0.0	0.0	0.0	0.0	0.0	0.0	0.0	0.0
Fats, oils	2.6	32.4	2.4	34.0	3.2	41.2	2.5	28.5	2.0	31.6	4.0	55.0
Sweets, sugars	0.0	0.2	0.1	1.2	0.1	1.3	0.1	0.6	0.0	0.4	0.1	0.7
Spices, condiments	0.0	0.0	0.0	0.1	0.0	0.0	0.0	0.1	0.0	0.1	0.0	0.0
Beverages	0.0	0.5	0.0	0.6	0.0	0.1	0.1	1.2	0.1	1.5	0.0	0.5
Savory snacks	0.0	0.0	0.0	0.0	0.0	0.0	0.0	0.0	0.0	0.0	0.0	0.0
Other	0.4	4.5	0.0	0.0	0.3	3.2	0.1	0.7	0.0	0.5	0.0	0.0
Total	8.1	100.0	7.2	100.0	7.9	100.0	8.8	100.0	6.4	100.0	7.3	100.0

OMN, omnivorous diet; VGT, vegetarian diet; VGN, vegan diet.

Table S21: Mean daily vitamin E (mg) intake and percentages of total intake by food groups in children and adults

	Children						Adults					
	OMN n=26		VGT n=18		VGN n=29		OMN n=32		VGT n=27		VGN n=34	
Food group	Mean	%	Mean	%	Mean	%	Mean	%	Mean	%	Mean	%
Cereals	1.1	16.9	1.1	12.0	1.3	13.1	1.3	11.4	1.6	11.3	1.6	10.1
Roots, tubers	0.0	0.8	0.1	0.9	0.1	1.1	0.2	1.5	0.1	0.7	0.1	0.8
Pulses, seeds, nuts	0.2	2.5	1.0	11.1	1.6	16.4	0.7	6.4	2.6	17.5	3.1	19.0
Plant-based dairy alternatives	0.3	5.4	1.1	13.1	2.3	22.9	0.3	2.9	1.4	9.3	2.6	16.0
Milk, dairy products	0.4	6.4	0.5	5.6	0.0	0.0	0.4	3.7	0.4	2.4	0.0	0.0
Eggs	0.3	3.9	0.2	1.8	0.0	0.0	0.6	5.2	0.4	2.7	0.0	0.0
Fish	0.4	6.6	0.2	2.0	0.0	0.0	0.8	7.0	0.3	2.3	0.0	0.0
Meat	0.3	4.8	0.0	0.3	0.0	0.0	0.8	7.0	0.0	0.1	0.0	0.0
Vegetables	0.5	8.3	0.9	10.4	0.5	5.3	1.1	9.6	2.3	15.8	1.6	10.1
Fruits	0.6	8.5	1.0	11.0	0.8	7.9	1.0	9.0	1.0	6.6	1.2	7.7
Fats, oils	1.8	27.2	2.3	25.8	2.7	27.1	3.1	26.5	3.2	21.7	4.4	27.2
Sweets, sugars	0.1	2.2	0.3	2.9	0.3	3.2	0.3	2.7	0.2	1.4	0.5	2.8
Spices, condiments	0.2	2.4	0.1	1.7	0.1	1.5	0.6	5.2	0.6	4.0	0.4	2.5
Beverages	0.1	0.8	0.0	0.2	0.0	0.3	0.1	0.9	0.2	1.1	0.2	1.1
Savory snacks	0.1	0.9	0.0	0.4	0.0	0.3	0.1	0.9	0.4	3.0	0.4	2.5
Other	0.2	2.5	0.1	0.8	0.1	0.8	0.0	0.1	0.0	0.0	0.0	0.3
Total	6.5	100.0	8.7	100.0	10.0	100.0	11.7	100.0	14.6	100.0	16.2	100.0

OMN, omnivorous diet; VGT, vegetarian diet; VGN, vegan diet.

Table S22: Mean daily vitamin K (µg) intake and percentages of total intake by food groups in children and adults

	Children						Adults					
	OMN n=26		VGT n=18		VGN n=29		OMN n=32		VGT n=27		VGN n=34	
Food group	Mean	%	Mean	%	Mean	%	Mean	%	Mean	%	Mean	%
Cereals	7.1	9.9	4.7	6.2	5.2	6.4	5.9	4.4	7.6	3.9	9.0	4.6
Roots, tubers	0.7	0.9	0.8	1.0	1.0	1.2	1.5	1.2	0.9	0.5	1.1	0.6
Pulses, seeds, nuts	3.9	5.4	10.0	13.1	8.8	10.8	7.8	5.9	20.5	10.6	16.1	8.2
Plant-based dairy alternatives	0.8	1.1	6.8	8.9	16.1	19.8	2.7	2.1	7.7	4.0	17.2	8.7
Milk, dairy products	4.0	5.6	3.9	5.1	0.0	0.0	5.0	3.7	5.0	2.6	0.0	0.0
Eggs	0.1	0.2	0.1	0.1	0.0	0.0	0.6	0.4	0.3	0.2	0.0	0.0
Fish	1.6	2.3	0.2	0.3	0.0	0.0	1.9	1.4	0.7	0.3	0.0	0.0
Meat	7.8	11.0	0.1	0.2	0.0	0.0	18.4	13.8	0.0	0.0	0.0	0.0
Vegetables	26.5	37.2	24.1	31.5	25.8	31.7	58.5	43.9	116.5	60.3	109.8	55.8
Fruits	5.7	8.0	9.2	12.0	6.5	7.9	8.8	6.6	7.2	3.7	12.0	6.1
Fats, oils	10.2	14.4	13.9	18.2	15.4	18.9	15.4	11.6	18.0	9.3	21.9	11.1
Sweets, sugars	0.9	1.3	1.3	1.7	1.2	1.4	1.4	1.0	1.2	0.6	2.0	1.0
Spices, condiments	0.9	1.3	0.9	1.2	0.8	0.9	4.1	3.0	5.4	2.8	4.8	2.4
Beverages	0.4	0.5	0.2	0.3	0.3	0.3	1.1	0.8	1.4	0.7	2.1	1.1
Savory snacks	0.1	0.2	0.0	0.0	0.1	0.1	0.2	0.1	0.7	0.4	0.7	0.4
Other	0.5	0.7	0.2	0.2	0.3	0.4	0.1	0.1	0.0	0.0	0.1	0.0
Total	71.2	100.0	76.4	100.0	81.4	100.0	133.3	100.0	193.1	100.0	196.9	100.0

OMN, omnivorous diet; VGT, vegetarian diet; VGN, vegan diet.

Table S23: Mean daily calcium (mg) intake and percentages of total intake by food groups in children and adults

	Children						Adults					
	OMN n=26		VGT n=18		VGN n=29		OMN n=32		VGT n=27		VGN n=34	
Food group	Mean	%	Mean	%	Mean	%	Mean	%	Mean	%	Mean	%
Cereals	36.8	4.7	33.9	4.0	35.2	4.6	48.9	4.8	52.4	5.1	54.2	5.7
Roots, tubers	3.9	0.5	3.6	0.4	3.2	0.4	4.4	0.4	5.4	0.5	5.8	0.6
Pulses, seeds, nuts	14.3	1.8	42.1	4.9	76.0	10.0	24.2	2.4	125.1	12.1	243.4	25.8
Plant-based dairy alternatives	60.2	7.7	255.9	30.0	558.0	73.2	67.8	6.7	238.5	23.1	436.3	46.2
Milk, dairy products	545.2	69.6	388.5	45.6	0.0	0.0	616.5	60.9	343.8	33.3	0.0	0.0
Eggs	7.6	1.0	4.8	0.6	0.0	0.0	17.1	1.7	11.2	1.1	0.0	0.0
Fish	8.2	1.0	5.5	0.6	0.0	0.0	13.1	1.3	20.0	1.9	0.0	0.0
Meat	7.6	1.0	0.4	0.1	0.0	0.0	15.3	1.5	0.2	0.0	0.0	0.0
Vegetables	23.0	2.9	29.6	3.5	26.0	3.4	49.7	4.9	86.1	8.3	75.8	8.0
Fruits	22.1	2.8	38.0	4.5	24.8	3.3	28.0	2.8	33.7	3.3	45.2	4.8
Fats, oils	1.4	0.2	1.0	0.1	0.7	0.1	1.9	0.2	1.9	0.2	1.0	0.1
Sweets, sugars	10.4	1.3	12.7	1.5	4.9	0.6	40.2	4.0	38.1	3.7	12.0	1.3
Spices, condiments	4.4	0.6	4.9	0.6	4.7	0.6	11.2	1.1	15.3	1.5	10.1	1.1
Beverages	10.3	1.3	13.5	1.6	12.6	1.7	34.3	3.4	41.4	4.0	35.1	3.7
Savory snacks	0.8	0.1	0.4	0.0	0.1	0.0	0.8	0.1	2.7	0.3	2.4	0.3
Other	26.6	3.4	17.6	2.1	16.3	2.1	39.4	3.9	17.6	1.7	22.2	2.3
Total	782.8	100.0	852.3	100.0	762.5	100.0	1012.7	100.0	1033.5	100.0	943.5	100.0

OMN, omnivorous diet; VGT, vegetarian diet; VGN, vegan diet.

Table S24: Mean daily iodine (µg) intake and percentages of total intake by food groups in children and adults

	Children						Adults					
	OMN n=26		VGT n=18		VGN n=29		OMN n=32		VGT n=27		VGN n=34	
Food group	Mean	%	Mean	%	Mean	%	Mean	%	Mean	%	Mean	%
Cereals	21.1	14.1	23.9	16.6	23.2	20.8	30.7	13.8	37.4	23.4	36.6	30.3
Roots, tubers	0.7	0.5	0.6	0.4	1.4	1.3	1.7	0.8	0.8	0.5	2.5	2.0
Pulses, seeds, nuts	0.6	0.4	3.9	2.7	1.8	1.6	2.6	1.2	4.6	2.9	3.6	3.0
Plant-based dairy alternatives	8.5	5.7	28.1	19.4	44.3	39.8	9.7	4.4	23.9	14.9	26.9	22.2
Milk, dairy products	60.3	40.2	40.9	28.3	0.0	0.0	50.3	22.6	18.7	11.7	0.0	0.0
Eggs	5.6	3.7	3.1	2.1	0.0	0.0	12.7	5.7	8.0	5.0	0.0	0.0
Fish	10.5	7.0	7.5	5.2	0.0	0.0	20.6	9.2	9.9	6.2	0.0	0.0
Meat	5.3	3.5	0.2	0.2	0.0	0.0	11.3	5.1	0.1	0.0	0.0	0.0
Vegetables	1.3	0.9	2.9	2.0	14.4	13.0	11.6	5.2	8.0	5.0	10.7	8.8
Fruits	1.4	0.9	2.0	1.4	1.8	1.6	1.5	0.7	1.3	0.8	1.9	1.6
Fats, oils	1.2	0.8	0.3	0.2	0.0	0.0	1.0	0.4	1.0	0.6	0.0	0.0
Sweets, sugars	2.2	1.5	3.3	2.3	1.2	1.1	6.4	2.9	4.7	3.0	2.3	1.9
Spices, condiments	27.1	18.1	26.4	18.3	21.9	19.7	56.1	25.2	34.5	21.5	30.9	25.5
Beverages	0.9	0.6	0.9	0.6	0.7	0.6	4.3	1.9	6.5	4.0	5.2	4.3
Savory snacks	0.0	0.0	0.0	0.0	0.0	0.0	0.0	0.0	0.0	0.0	0.0	0.0
Other	3.1	2.1	0.7	0.5	0.6	0.5	2.1	1.0	0.6	0.4	0.2	0.1
Total	150.0	100.0	144.6	100.0	111.4	100.0	222.8	100.0	160.1	100.0	120.8	100.0

OMN, omnivorous diet; VGT, vegetarian diet; VGN, vegan diet.

Table S25: Mean daily iron (mg) intake and percentages of total intake by food groups in children and adults

	Children						Adults					
	OMN n=26		VGT n=18		VGN n=29		OMN n=32		VGT n=27		VGN n=34	
Food group	Mean	%	Mean	%	Mean	%	Mean	%	Mean	%	Mean	%
Cereals	2.9	40.9	3.2	32.9	3.5	31.8	3.9	33.9	4.8	30.2	4.7	26.8
Roots, tubers	0.4	4.9	0.3	3.0	0.3	2.6	0.5	4.1	0.3	2.2	0.5	2.6
Pulses, seeds, nuts	0.5	6.5	1.9	19.3	2.1	19.3	1.1	9.8	4.2	26.5	5.3	29.8
Plant-based dairy alternatives	0.3	3.8	1.6	16.3	3.0	27.8	0.5	4.5	1.7	10.4	2.7	15.5
Milk, dairy products	0.2	3.3	0.2	1.6	0.0	0.0	0.3	2.5	0.2	1.0	0.0	0.0
Eggs	0.2	3.1	0.1	1.4	0.0	0.0	0.5	4.4	0.3	2.1	0.0	0.0
Fish	0.2	2.3	0.1	0.8	0.0	0.0	0.4	3.1	0.2	1.4	0.0	0.0
Meat	0.8	10.4	0.1	0.8	0.0	0.0	1.4	12.4	0.0	0.3	0.0	0.0
Vegetables	0.4	5.7	0.6	5.9	0.5	4.6	0.9	7.8	1.7	10.6	1.4	7.8
Fruits	0.7	9.2	1.2	11.7	0.8	7.7	0.8	6.7	0.7	4.5	1.2	6.6
Fats, oils	0.0	0.2	0.0	0.1	0.0	0.2	0.0	0.2	0.0	0.1	0.0	0.2
Sweets, sugars	0.2	2.1	0.3	3.0	0.2	2.2	0.4	3.8	0.5	3.3	0.6	3.5
Spices, condiments	0.1	1.1	0.1	0.9	0.1	1.0	0.2	1.8	0.4	2.3	0.3	1.6
Beverages	0.1	1.1	0.1	0.6	0.1	1.1	0.4	3.8	0.5	3.1	0.5	3.1
Savory snacks	0.0	0.4	0.0	0.1	0.0	0.1	0.0	0.3	0.2	1.1	0.2	0.9
Other	0.3	4.8	0.1	1.5	0.2	1.6	0.1	0.9	0.1	0.9	0.3	1.7
Total	7.2	100.0	9.9	100.0	10.9	100.0	11.6	100.0	15.9	100.0	17.7	100.0

OMN, omnivorous diet; VGT, vegetarian diet; VGN, vegan diet.

Table S26: Mean daily magnesium (mg) intake and percentages of total intake by food groups in children and adults

	Children						Adults					
	OMN n=26		VGT n=18		VGN n=29		OMN n=32		VGT n=27		VGN n=34	
Food group	Mean	%	Mean	%	Mean	%	Mean	%	Mean	%	Mean	%
Cereals	79.1	34.4	92.0	30.3	98.4	29.9	107.4	27.7	129.1	26.7	127.4	23.9
Roots, tubers	12.7	5.5	10.7	3.5	10.2	3.1	16.9	4.3	12.7	2.6	16.4	3.1
Pulses, seeds, nuts	14.7	6.4	49.4	16.3	62.8	19.1	43.9	11.3	122.0	25.3	147.4	27.6
Plant-based dairy alternatives	7.9	3.4	47.8	15.8	100.0	30.4	12.2	3.1	45.1	9.3	84.2	15.8
Milk, dairy products	49.0	21.3	33.2	10.9	0.0	0.0	41.2	10.6	17.9	3.7	0.0	0.0
Eggs	1.8	0.8	1.1	0.4	0.0	0.0	3.9	1.0	2.6	0.5	0.0	0.0
Fish	5.5	2.4	2.9	1.0	0.0	0.0	10.9	2.8	5.3	1.1	0.0	0.0
Meat	10.6	4.6	0.8	0.3	0.0	0.0	26.3	6.8	0.4	0.1	0.0	0.0
Vegetables	13.5	5.9	17.8	5.9	15.4	4.7	28.4	7.3	50.1	10.4	41.2	7.7
Fruits	17.3	7.5	34.2	11.3	24.7	7.5	21.7	5.6	25.1	5.2	36.0	6.8
Fats, oils	0.3	0.1	0.3	0.1	0.3	0.1	0.4	0.1	0.3	0.1	0.4	0.1
Sweets, sugars	4.5	1.9	5.7	1.9	7.4	2.3	13.2	3.4	12.8	2.6	18.4	3.4
Spices, condiments	1.7	0.7	1.6	0.5	1.9	0.6	3.4	0.9	3.7	0.8	3.5	0.7
Beverages	4.2	1.8	3.0	1.0	5.2	1.6	52.6	13.5	49.4	10.2	49.3	9.2
Savory snacks	1.0	0.4	0.6	0.2	0.3	0.1	0.8	0.2	4.8	1.0	4.2	0.8
Other	6.0	2.6	2.1	0.7	2.6	0.8	5.3	1.4	1.8	0.4	4.8	0.9
Total	229.8	100.0	303.3	100.0	329.1	100.0	388.4	100.0	483.2	100.0	533.0	100.0

OMN, omnivorous diet; VGT, vegetarian diet; VGN, vegan diet.

Table S27: Mean daily phosphorus (mg) intake and percentages of total intake by food groups in children and adults

	Children						Adults					
	OMN n=26		VGT n=18		VGN n=29		OMN n=32		VGT n=27		VGN n=34	
Food group	Mean	%	Mean	%	Mean	%	Mean	%	Mean	%	Mean	%
Cereals	251.2	24.9	285.2	27.8	306.9	36.4	352.8	22.9	410.0	28.4	403.3	30.2
Roots, tubers	24.2	2.4	20.4	2.0	20.4	2.4	32.0	2.1	24.1	1.7	33.8	2.5
Pulses, seeds, nuts	37.2	3.7	124.9	12.2	154.9	18.4	97.8	6.3	291.6	20.2	394.6	29.5
Plant-based dairy alternatives	25.4	2.5	119.1	11.6	241.6	28.6	35.5	2.3	116.3	8.0	206.2	15.4
Milk, dairy products	401.4	39.8	287.0	28.0	0.0	0.0	433.8	28.2	221.1	15.3	0.0	0.0
Eggs	28.0	2.8	17.5	1.7	0.0	0.0	62.6	4.1	41.0	2.8	0.1	0.0
Fish	44.2	4.4	26.7	2.6	0.0	0.0	93.1	6.0	42.8	3.0	0.0	0.0
Meat	78.1	7.8	6.4	0.6	0.0	0.0	193.8	12.6	3.5	0.2	0.0	0.0
Vegetables	35.0	3.5	53.3	5.2	40.2	4.8	69.4	4.5	127.8	8.8	111.2	8.3
Fruits	25.3	2.5	43.8	4.3	32.9	3.9	30.3	2.0	31.4	2.2	45.6	3.4
Fats, oils	1.3	0.1	0.8	0.1	0.2	0.0	2.1	0.1	2.1	0.1	0.8	0.1
Sweets, sugars	13.0	1.3	20.0	1.9	13.4	1.6	43.3	2.8	31.9	2.2	28.4	2.1
Spices, condiments	4.1	0.4	4.1	0.4	4.5	0.5	9.4	0.6	12.3	0.9	8.9	0.7
Beverages	7.7	0.8	7.3	0.7	7.3	0.9	54.4	3.5	56.9	3.9	56.5	4.2
Savory snacks	2.8	0.3	1.7	0.2	0.8	0.1	2.5	0.2	14.1	1.0	12.3	0.9
Other	28.9	2.9	8.3	0.8	20.3	2.4	27.2	1.8	18.0	1.2	33.8	2.5
Total	1007.8	100.0	1026.4	100.0	843.3	100.0	1539.8	100.0	1444.9	100.0	1335.5	100.0

OMN, omnivorous diet; VGT, vegetarian diet; VGN, vegan diet.

Table S28: Mean daily potassium (mg) intake and percentages of total intake by food groups in children and adults

	Children						Adults					
	OMN n=26		VGT n=18		VGN n=29		OMN n=32		VGT n=27		VGN n=34	
Food group	Mean	%	Mean	%	Mean	%	Mean	%	Mean	%	Mean	%
Cereals	313.8	14.3	343.1	14.1	372.4	17.1	423.2	12.4	518.9	14.1	521.2	13.3
Roots, tubers	213.9	9.8	178.4	7.3	169.2	7.8	292.4	8.6	221.6	6.0	282.6	7.2
Pulses, seeds, nuts	60.1	2.7	259.9	10.7	289.9	13.3	140.5	4.1	557.8	15.2	650.6	16.6
Plant-based dairy alternatives	23.8	1.1	200.3	8.2	504.2	23.2	46.5	1.4	188.7	5.1	451.0	11.5
Milk, dairy products	616.4	28.1	401.5	16.5	0.0	0.0	444.4	13.0	138.5	3.8	0.0	0.0
Eggs	18.0	0.8	11.8	0.5	0.0	0.0	38.7	1.1	26.3	0.7	0.0	0.0
Fish	84.5	3.9	48.9	2.0	0.0	0.0	150.2	4.4	70.9	1.9	0.0	0.0
Meat	134.8	6.2	11.0	0.5	0.0	0.0	362.5	10.6	5.8	0.2	0.0	0.0
Vegetables	302.0	13.8	403.5	16.6	312.3	14.3	581.2	17.0	999.2	27.2	840.8	21.4
Fruits	250.5	11.4	449.3	18.4	354.7	16.3	285.3	8.4	325.9	8.9	482.4	12.3
Fats, oils	1.0	0.0	0.7	0.0	0.2	0.0	1.7	0.1	1.7	0.0	1.1	0.0
Sweets, sugars	35.1	1.6	49.5	2.0	47.1	2.2	93.0	2.7	83.3	2.3	97.3	2.5
Spices, condiments	17.3	0.8	11.3	0.5	22.8	1.0	16.9	0.5	19.4	0.5	18.2	0.5
Beverages	55.4	2.5	36.4	1.5	74.4	3.4	458.1	13.4	432.8	11.8	457.1	11.7
Savory snacks	13.3	0.6	2.1	0.1	5.2	0.2	12.0	0.4	71.0	1.9	73.9	1.9
Other	51.0	2.3	29.9	1.2	23.9	1.1	64.1	1.9	18.5	0.5	46.7	1.2
Total	2190.9	100.0	2437.6	100.0	2176.1	100.0	3410.9	100.0	3680.3	100.0	3922.8	100.0

OMN, omnivorous diet; VGT, vegetarian diet; VGN, vegan diet.

Table S29: Mean daily selenium (µg) intake and percentages of total intake by food groups in children and adults

	Children						Adults					
	OMN n=26		VGT n=18		VGN n=29		OMN n=32		VGT n=27		VGN n=34	
Food group	Mean	%	Mean	%	Mean	%	Mean	%	Mean	%	Mean	%
Cereals	9.2	20.6	9.8	27.2	10.6	41.6	13.6	13.9	14.3	21.6	14.1	33.3
Roots, tubers	0.4	0.8	0.3	0.8	0.3	1.1	0.5	0.5	0.4	0.6	0.5	1.1
Pulses, seeds, nuts	1.7	3.7	3.3	9.0	5.5	21.3	21.0	21.5	23.7	35.7	16.7	39.4
Plant-based dairy alternatives	0.6	1.4	3.7	10.3	7.2	28.0	0.8	0.9	3.1	4.6	6.1	14.5
Milk, dairy products	13.0	29.1	9.0	25.0	0.0	0.0	14.3	14.7	7.5	11.4	0.0	0.0
Eggs	4.5	10.0	2.8	7.9	0.0	0.0	9.8	10.1	6.5	9.8	0.0	0.0
Fish	5.3	11.9	3.5	9.6	0.0	0.0	11.5	11.8	4.7	7.1	0.0	0.0
Meat	7.7	17.2	0.6	1.8	0.0	0.0	21.2	21.8	0.3	0.5	0.0	0.0
Vegetables	0.4	1.0	0.7	2.0	0.4	1.7	1.3	1.3	2.2	3.3	1.7	3.9
Fruits	0.5	1.1	0.5	1.5	0.5	1.9	0.7	0.7	0.7	1.0	0.9	2.0
Fats, oils	0.0	0.0	0.0	0.0	0.0	0.0	0.0	0.0	0.0	0.0	0.0	0.0
Sweets, sugars	0.5	1.2	1.2	3.2	0.6	2.2	1.1	1.1	1.3	1.9	0.8	1.8
Spices, condiments	0.2	0.4	0.2	0.6	0.1	0.4	0.7	0.7	0.5	0.8	0.3	0.8
Beverages	0.2	0.4	0.3	0.7	0.2	0.7	0.6	0.6	0.6	1.0	0.5	1.3
Savory snacks	0.1	0.2	0.0	0.1	0.0	0.1	0.1	0.1	0.4	0.6	0.3	0.7
Other	0.5	1.0	0.1	0.3	0.2	0.9	0.4	0.4	0.1	0.2	0.5	1.1
Total	44.8	100.0	36.2	100.0	25.6	100.0	97.5	100.0	66.4	100.0	42.4	100.0

OMN, omnivorous diet; VGT, vegetarian diet; VGN, vegan diet.

Table S30: Mean daily zinc (mg) intake and percentages of total intake by food groups in children and adults

	Children						Adults					
	OMN n=26		VGT n=18		VGN n=29		OMN n=32		VGT n=27		VGN n=34	
Food group	Mean	%	Mean	%	Mean	%	Mean	%	Mean	%	Mean	%
Cereals	2.1	28.6	2.4	32.1	2.6	38.2	2.8	24.9	3.4	31.8	3.3	32.3
Roots, tubers	0.2	2.3	0.1	1.9	0.1	2.1	0.2	1.9	0.2	1.5	0.2	2.3
Pulses, seeds, nuts	0.3	4.4	1.0	13.4	1.2	18.5	0.9	7.6	2.3	20.9	3.0	29.0
Plant-based dairy alternatives	0.2	2.7	0.7	10.0	1.5	22.1	0.2	2.2	0.8	6.9	1.3	13.0
Milk, dairy products	2.1	28.5	1.5	20.8	0.0	0.0	2.5	21.8	1.5	13.8	0.0	0.0
Eggs	0.2	2.5	0.1	1.6	0.0	0.0	0.4	3.6	0.3	2.5	0.0	0.0
Fish	0.2	2.1	0.1	0.9	0.0	0.0	0.3	2.7	0.2	1.7	0.0	0.0
Meat	1.3	17.7	0.1	1.5	0.0	0.0	2.6	22.4	0.1	0.6	0.0	0.0
Vegetables	0.2	3.4	0.4	5.6	0.3	4.2	0.5	4.5	0.9	8.1	0.7	7.1
Fruits	0.2	2.1	0.3	4.2	0.2	3.0	0.2	1.5	0.2	1.6	0.3	2.6
Fats, oils	0.0	0.1	0.0	0.0	0.0	0.0	0.0	0.1	0.0	0.1	0.0	0.0
Sweets, sugars	0.1	1.3	0.1	1.8	0.1	1.7	0.3	2.4	0.2	2.0	0.3	2.5
Spices, condiments	0.0	0.3	0.0	0.3	0.0	0.3	0.0	0.4	0.1	0.5	0.0	0.4
Beverages	0.1	1.1	0.0	0.6	0.0	0.6	0.3	2.5	0.2	1.7	0.2	1.8
Savory snacks	0.0	0.3	0.0	0.1	0.0	0.1	0.0	0.2	0.1	0.8	0.1	0.7
Other	0.2	2.8	0.4	5.3	0.6	9.2	0.2	1.4	0.6	5.6	0.9	8.4
Total	7.3	100.0	7.4	100.0	6.7	100.0	11.4	100.0	10.8	100.0	10.2	100.0

OMN, omnivorous diet; VGT, vegetarian diet; VGN, vegan diet.

Table S31: Mean daily linoleic acid (18:2 n-6, mg) intake and percentages of total intake by food groups in children and adults

	Children						Adults					
	OMN n=26		VGT n=18		VGN n=29		OMN n=32		VGT n=27		VGN n=34	
Food group	Mean	%	Mean	%	Mean	%	Mean	%	Mean	%	Mean	%
Cereals	1508.9	28.4	1515.1	19.9	1821.7	17.6	1999.3	18.0	2215.6	14.5	2331.6	12.8
Roots, tubers	24.3	0.5	33.7	0.4	95.2	0.9	121.2	1.1	73.7	0.5	69.6	0.4
Pulses, seeds, nuts	455.6	8.6	1638.6	21.5	2595.8	25.1	1846.1	16.7	4876.2	31.9	6374.9	35.0
Plant-based dairy alternatives	284.6	5.4	1485.7	19.5	3454.1	33.4	546.5	4.9	1642.9	10.7	3575.6	19.6
Milk, dairy products	221.7	4.2	293.4	3.8	0.0	0.0	303.3	2.7	277.3	1.8	0.0	0.0
Eggs	134.8	2.5	93.8	1.2	0.0	0.0	377.6	3.4	246.5	1.6	0.3	0.0
Fish	302.6	5.7	41.9	0.5	0.0	0.0	372.2	3.4	151.4	1.0	0.0	0.0
Meat	510.4	9.6	12.9	0.2	0.0	0.0	1299.4	11.7	7.3	0.0	0.0	0.0
Vegetables	79.0	1.5	323.0	4.2	135.4	1.3	214.9	1.9	514.1	3.4	392.2	2.2
Fruits	80.0	1.5	130.2	1.7	117.8	1.1	152.2	1.4	294.7	1.9	273.5	1.5
Fats, oils	1134.7	21.4	1617.3	21.2	1660.4	16.1	2692.9	24.3	3327.9	21.7	3661.8	20.1
Sweets, sugars	152.9	2.9	249.4	3.3	291.9	2.8	369.0	3.3	221.6	1.4	388.7	2.1
Spices, condiments	114.9	2.2	120.7	1.6	34.7	0.3	620.0	5.6	686.3	4.5	367.0	2.0
Beverages	10.0	0.2	5.1	0.1	15.4	0.1	13.2	0.1	72.6	0.5	56.2	0.3
Savory snacks	101.6	1.9	40.6	0.5	47.2	0.5	137.3	1.2	681.0	4.5	659.4	3.6
Other	194.2	3.7	30.5	0.4	60.2	0.6	17.3	0.2	12.5	0.1	49.3	0.3
Total	5310.1	100.0	7631.9	100.0	10 329.8	100.0	11 082.4	100.0	15 301.7	100.0	18 200.2	100.0

OMN, omnivorous diet; VGT, vegetarian diet; VGN, vegan diet.

Table S32: Mean daily a-linolenic acid (18:3 n-3, mg) intake and percentages of total intake by food groups in children and adults

	Children						Adults					
	OMN n=26		VGT n=18		VGN n=29		OMN n=32		VGT n=27		VGN n=34	
Food group	Mean	%	Mean	%	Mean	%	Mean	%	Mean	%	Mean	%
Cereals	289.7	21.3	272.9	12.7	318.3	12.0	323.1	12.4	386.4	11.0	382.7	7.8
Roots, tubers	15.0	1.1	13.4	0.6	24.6	0.9	40.3	1.6	22.3	0.6	21.7	0.4
Pulses, seeds, nuts	114.9	8.5	358.8	16.7	395.9	14.9	257.7	9.9	625.5	17.8	1084.9	22.1
Plant-based dairy alternatives	57.5	4.2	401.3	18.6	873.0	32.8	193.5	7.5	490.4	14.0	1007.1	20.5
Milk, dairy products	50.3	3.7	79.7	3.7	0.0	0.0	103.2	4.0	100.6	2.9	0.0	0.0
Eggs	10.2	0.7	5.5	0.3	0.0	0.0	43.6	1.7	26.0	0.7	0.0	0.0
Fish	112.8	8.3	10.3	0.5	0.0	0.0	133.3	5.1	38.4	1.1	0.0	0.0
Meat	87.7	6.4	5.3	0.2	0.0	0.0	203.3	7.8	3.3	0.1	0.0	0.0
Vegetables	27.8	2.0	112.3	5.2	55.2	2.1	79.5	3.1	198.9	5.7	158.0	3.2
Fruits	33.8	2.5	75.3	3.5	49.0	1.8	66.6	2.6	67.7	1.9	107.3	2.2
Fats, oils	438.6	32.3	710.7	33.0	847.9	31.9	861.4	33.2	1145.6	32.6	1740.7	35.5
Sweets, sugars	41.0	3.0	42.6	2.0	61.6	2.3	63.7	2.5	30.3	0.9	108.2	2.2
Spices, condiments	29.0	2.1	45.1	2.1	8.2	0.3	198.1	7.6	207.2	5.9	133.6	2.7
Beverages	5.5	0.4	1.6	0.1	5.5	0.2	4.9	0.2	26.6	0.8	26.9	0.5
Savory snacks	23.0	1.7	10.7	0.5	9.1	0.3	19.6	0.8	139.5	4.0	132.4	2.7
Other	22.5	1.7	7.2	0.3	11.8	0.4	3.5	0.1	2.6	0.1	3.8	0.1
Total	1359.3	100.0	2152.7	100.0	2660.0	100.0	2595.2	100.0	3511.1	100.0	4907.2	100.0

OMN, omnivorous diet; VGT, vegetarian diet; VGN, vegan diet.

Table S33: Mean daily eicosapentaenoic acid (20:5 n-3, mg) intake and percentages of total intake by food groups in children and adults

	Children						Adults					
	OMN n=26		VGT n=18		VGN n=29		OMN n=32		VGT n=27		VGN n=34	
Food group	Mean	%	Mean	%	Mean	%	Mean	%	Mean	%	Mean	%
Cereals	0.0	0.0	0.0	0.0	0.0	-	0.0	0.0	0.0	0.0	0.0	0.0
Roots, tubers	0.0	0.0	0.0	0.0	0.0	-	0.0	0.0	0.0	0.0	0.0	0.0
Pulses, seeds, nuts	0.0	0.0	0.0	0.0	0.0	-	0.0	0.0	0.0	0.0	0.0	0.0
Plant-based dairy alternatives	0.0	0.0	0.0	0.0	0.0	-	0.0	0.0	0.0	0.0	0.0	0.0
Milk, dairy products	0.0	0.0	0.0	0.1	0.0	-	0.1	0.0	0.1	0.1	0.0	0.0
Eggs	0.0	0.0	0.0	0.0	0.0	-	0.0	0.0	0.0	0.0	0.0	0.0
Fish	56.9	93.0	29.1	97.4	0.0	-	171.0	94.3	54.0	98.7	0.0	0.0
Meat	4.3	7.0	0.8	2.5	0.0	-	10.3	5.7	0.4	0.8	0.0	0.0
Vegetables	0.0	0.0	0.0	0.0	0.0	-	0.0	0.0	0.0	0.0	0.0	0.0
Fruits	0.0	0.0	0.0	0.0	0.0	-	0.0	0.0	0.0	0.0	0.0	0.0
Fats, oils	0.0	0.0	0.0	0.0	0.0	-	0.0	0.0	0.0	0.0	0.0	0.0
Sweets, sugars	0.0	0.0	0.0	0.0	0.0	-	0.0	0.0	0.0	0.0	0.0	0.0
Spices, condiments	0.0	0.0	0.0	0.0	0.0	-	0.0	0.0	0.2	0.4	0.0	100.0
Beverages	0.0	0.0	0.0	0.0	0.0	-	0.0	0.0	0.0	0.0	0.0	0.0
Savory snacks	0.0	0.0	0.0	0.0	0.0	-	0.0	0.0	0.0	0.0	0.0	0.0
Other	0.0	0.0	0.0	0.0	0.0	-	0.0	0.0	0.0	0.0	0.0	0.0
Total	61.2	100.0	29.9	100.0	0.0	na	181.4	100.0	54.7	100.0	0.0	100.0

OMN, omnivorous diet; VGT, vegetarian diet; VGN, vegan diet.

na, no percentages calculated for EPA with zero intake in the VGN group (children).

Table S34: Mean daily docosahexaenoic acid (22:6 n-3, mg) intake and percentages of total intake by food groups in children and adults

	Children						Adults					
	OMN n=26		VGT n=18		VGN n=29		OMN n=32		VGT n=27		VGN n=34	
Food group	Mean	%	Mean	%	Mean	%	Mean	%	Mean	%	Mean	%
Cereals	0.5	0.3	0.6	0.6	0.1	100.0	0.7	0.1	0.8	0.5	0.9	94.3
Roots, tubers	0.0	0.0	0.0	0.0	0.0	0.0	0.0	0.0	0.0	0.0	0.0	0.0
Pulses, seeds, nuts	0.0	0.0	0.0	0.0	0.0	0.0	0.0	0.0	0.0	0.0	0.0	0.0
Plant-based dairy alternatives	0.0	0.0	0.0	0.0	0.0	0.0	0.0	0.0	0.0	0.0	0.0	0.0
Milk, dairy products	0.0	0.0	0.0	0.0	0.0	0.0	0.0	0.0	0.0	0.0	0.0	0.0
Eggs	14.4	8.4	9.7	10.1	0.0	0.0	33.6	6.5	22.3	13.7	0.0	3.2
Fish	153.2	88.7	84.8	87.8	0.0	0.0	467.7	91.0	138.4	85.2	0.0	0.0
Meat	4.3	2.5	0.1	0.1	0.0	0.0	10.9	2.1	0.0	0.0	0.0	0.0
Vegetables	0.0	0.0	0.0	0.0	0.0	0.0	0.0	0.0	0.0	0.0	0.0	1.5
Fruits	0.0	0.0	0.0	0.0	0.0	0.0	0.0	0.0	0.0	0.0	0.0	0.0
Fats, oils	0.0	0.0	0.0	0.0	0.0	0.0	0.0	0.0	0.0	0.0	0.0	0.0
Sweets, sugars	0.3	0.2	0.9	1.0	0.0	0.0	0.4	0.1	0.4	0.3	0.0	1.0
Spices, condiments	0.0	0.0	0.4	0.4	0.0	0.0	0.6	0.1	0.5	0.3	0.0	0.0
Beverages	0.0	0.0	0.0	0.0	0.0	0.0	0.0	0.0	0.0	0.0	0.0	0.0
Savory snacks	0.0	0.0	0.0	0.0	0.0	0.0	0.0	0.0	0.0	0.0	0.0	0.0
Other	0.0	0.0	0.0	0.0	0.0	0.0	0.0	0.0	0.0	0.0	0.0	0.0
Total	172.7	100.0	96.5	100.0	0.1	100.0	514.0	100.0	162.4	100.0	1.0	100.0

OMN, omnivorous diet; VGT, vegetarian diet; VGN, vegan diet.
